# Supplementary material for: The role of water in host-guest interaction
Source: Nat Commun. 2021 Jan 4;12:93. doi: 10.1038/s41467-020-20310-0 (PMC7782548; doi:10.1038/s41467-020-20310-0)
Supplement: Supplementary file 1 — Supplementary Information [file 41467_2020_20310_MOESM1_ESM.pdf]

Supplementary Information: The role of water in host-guest interaction

Valerio Rizzi[1,3], Luigi Bonati[2,3], Narjes Ansari[1,3], and Michele Parrinello[1,3,4,*]

[1]Department of Chemistry and Applied Biosciences, ETH Zurich, 8092 Zurich, Switzerland
[2]Department of Physics, ETH Zurich, 8092 Zurich, Switzerland
[3]Facoltà di Informatica, Istituto di Scienze Computazionali, Università della Svizzera Italiana, Via G. Buffi 13, 6900 Lugano, Switzerland
[4]Italian Institute of Technology, Via Morego 30, 16163 Genova, Italy
[*]michele.parrinello@phys.chem.ethz.ch

November 19, 2020

Supplementary Methods

We follow the plead from [1] where the authors advocated the use of standard setups with a prescribed set of and simulation parameters for benchmarking methods within identical conditions. We have taken the input files from the public repository at https://github.com/michellab/Sire-SAMPL5. This simulation setup was used by a number of research groups [2, 3, 4] to investigate ligand binding with different methodologies.

We perform the simulations with GROMACS 2019.4 [5] in combination with the PLUMED plugin 2.5.4 [6] and the Pytorch library 1.4 [7]. We use the GAFF force field [8] with RESP charges [9] and the TIP3P water model [10]. Our timestep is 2 fs and the temperature is set at 300 K via a velocity rescale thermostat [11] with time constant 0.1 ps. The simulation box is cubic with a side of about 40 Å and it contains 2100 water molecules in solution together with the host OAMe and the chosen guest molecule. Sodium ions are included to counterbalance excess charges. At every simulation step, the coordinates are aligned so that the z axis of the box coincides with the binding axis and the simulation box is centred on the virtual atom V_1.

In the following sections, we analyse the most relevant aspects of the strategy proposed in the paper.

The descriptor set

Our descriptor set **d** specialises in measuring the water presence around chosen points and includes two species. The first species is centred on a set of ligand atoms L and the second one on virtual atoms V along the binding axis. The L descriptors specialise in measuring the local water solvation in the vicinity of the ligand, while the V descriptors control the host's solvation.

We choose 4 points L for every guest molecule and 8 points V, starting from the centre of the lower phenyl rings of the host and upward, with a spacing of 2.5 Å, as indicated in Fig. 1 in the main text. The water coordination number (CN) of every point i is calculated with

$$\mathrm{d}_i^0 = \sum_j^{r_{ij}<r_{\mathrm{NL}}} \frac{1-\left(\frac{r_{ij}}{r_0}\right)^n}{1-\left(\frac{r_{ik}}{r_0}\right)^m} \tag{1}$$

where r_{ij} measures the distance between point i and the water oxygen atom j within a sphere of radius $r_{\mathrm{NL}} = 10\,\text{Å}$ and $r_0 = 2.5\,\text{Å}$.

The parameters n and m are crucial for tuning the descriptor's effectiveness in enhanced sampling applications. Every descriptor has to be sufficiently short range to focus on the important water molecules in the vicinity of i, but also sufficiently long range to make its derivative smooth with respect to the more distant water molecules. For $d_i \in \mathrm{L}$ we choose $n = 6$ and $m = 10$ that provide a good compromise between short range focus and long range outreach. For $d_i \in \mathrm{V}$, there is no electrostatic repulsion that prevents the water molecules from reaching $r_{ij} \approx 0$, so we choose $n = 2$ and $m = 6$ that produces a switching function with an analogous long range behaviour, but a softer core at short distances. The CNs are then normalised so that their values d_i lie between -1 and 1. For $d_i \in \mathrm{L}$, we normalise the descriptor by $d_i = d_i^0/2.5 - 1$, while for $d_i \in \mathrm{V}$ we normalise it by $d_i = d_i^0/2.8 - 1$.

Neural network architecture and training

The Deep-LDA strategy that we use in this paper is completely akin to the one presented in [12], so we refer the interested reader to that paper for more details. A tutorial of Deep-LDA is present at the following link https://github.com/luigibonati/data-driven-CVs. Our neural network (NN) architecture is sketched in Fig. 2 in the main text and it consists of a sequence of layers with $12, 10, 8, 6, 4$ nodes, with the rectified linear unit as activation function. Therefore $N_{\mathrm{d}} = 12$ and $N_{\mathrm{h}} = 4$.

The first layer takes as input short trajectories of the normalised descriptors $\mathbf{d}$ calculated in state B and U. Given the presence of the funnel restraint in the subsequent enhanced sampling simulations, in the state U simulations used for training we trap the ligand in a cylindrical volume above the host. For state B, we take as a starting configuration the binding pose provided by the standard setup with randomised velocities. Typically, for each state we collect 5 trajectories of 4 ns where the descriptors are printed every 0.25 ps. Out of the resulting 80000 configurations, we randomly select 25600, put them in small batches of 512 elements and feed them to the NN for training.

A regularisation is applied to the within class scatter matrix $\mathbf{S}_w$ defined in the main text so that it becomes $\mathbf{S}'_w = \mathbf{S}_w + \lambda \mathbf{I}$ with $\lambda = 0.05$. Maximising Fisher's ratio in Eq. 1 is equivalent to solving the generalised eigenvalue problem

$$\boldsymbol{S}_{\mathrm{b}} \boldsymbol{w}_i = v_i \boldsymbol{S}'_{\mathrm{w}} \boldsymbol{w}_i \quad \forall i = 1, 2, \tag{2}$$

where $\boldsymbol{S}_{\mathrm{w}}$ and $\boldsymbol{S}_{\mathrm{b}}$ are $N_{\mathrm{h}} \times N_{\mathrm{h}}$-dimensional matrices calculated on the last hidden layer $\mathbf{h}$. In this 2-class problem, the largest eigenvalue $v = v_1$ measures the separation between states B and U along direction $\boldsymbol{w} = \boldsymbol{w}_1$.

The loss function that we use for training the NN is

$$\mathcal{L} = -v \; - \; \alpha \, \frac{1}{1 + (s^2 - 1)^2} \; + \; \gamma \sum_i |\theta_i|^2 \tag{3}$$

where the first term is the LDA eigenvalue, the second term prevents the NN output s from becoming too narrow over the training data, and the third one is an L2 regularization over the weights θ_i of the network, with $\gamma = 10^{-5}$. We set $\alpha = 2/\lambda$.

We optimise the model with ADAM [13] using a learning rate of $2.5 \cdot 10^{-5}$. We stop the training when the model reaches convergence, that we define through the condition $v > 1.28/\lambda$. The converged LDA eigenvector $\boldsymbol{w}$ generates $s = \boldsymbol{w}^T \boldsymbol{h}$. Throughout the paper, we transform the NN output by $s_{\mathrm{w}} = s + s^3$. This transformation improves the behaviour of the Deep-LDA CV s_{w} in enhanced sampling, as it increases its width in the important input states B and U.

The Funnel restraint

In the enhanced sampling simulations, we use a funnel restraint [14] equivalent to the one previously employed by [4, 15] on the same system. The funnel limits the space available to the ligand in state U by confining it to a cylindrical volume above the binding site, as sketched in Fig. 1 in the main text. As the

ligand approaches the binding site, the funnel restraint becomes wider so that its presence does not affect the binding process itself.

We define s_z as the projection on the binding axis z of the center of the carbon atoms of each ligand and r its radial component. When $s_z > 10\,\text{Å}$, the funnel surface is a cylinder with radius $R_{\text{cyl}} = 2\,\text{Å}$ with its axis along the z direction. When $s_z < 10\,\text{Å}$, the funnel opens into an umbrella-like shape with a 45 degree angle whose surface is defined by $r = 12 - s_z$.

The force that pushes the ligand for displacements x away from the funnel's surface is harmonic $-k_F x$ with $k_F = 20$ kJ mol^{-1} Å^{-2}. A further harmonic restraint is applied on s_z to prevent the ligand from getting too far from the host reaching the upper boundary of the simulation box. The corresponding force is $-k_U(s_z - 18)$ for $s_z > 18$Å and $k_F = 40$ kJ mol^{-1} Å^{-2}.

During training, we set boundaries to state U so that the training configurations match the ones that occur in the subsequent enhanced sampling simulations. We activate the funnel described above and two additional restraints $-k_U(s_z - 18)$ for $s_z > 18$ Å and $-k_U(s_z - 14)$ for $s_z < 18$ Å, with $k_U = 20$ kJ mol^{-1} Å^{-2}.

Because of the funnel presence, the free energy difference between the bound and the true unbound state that we extract from enhanced sampling simulations needs a correction. It can be calculated from

$$\Delta G = -\frac{1}{\beta} \log \left(C^0 \pi R_{\text{cyl}}^2 \int_B \text{d}z \exp\left(-\beta(W(z) - W_U)\right) \right) \tag{4}$$

where $\beta = 1/k_B T$, $C^0 = 1/1660\text{Å}^{-3}$ is the standard concentration, z is the coordinate along the funnel's axis, $W(z)$ is the free energy along the funnel axis and W_U its reference value in state U. More precisely, we define W_U as the average free energy value in the interval $1.5\,\text{Å} < z < 1.8\,\text{Å}$. The integral is performed over the state B region that we define as $0.3\,\text{Å} < z < 0.8\,\text{Å}$.

Enhanced sampling simulations

We perform enhanced sampling with OPES [16], a recently developed evolution of Metadynamics that helps achieving a fast and robust convergence. For every guest molecule, we independently train 3 different Deep-LDA CVs: s_W^a, s_W^b and s_W^c. With each one of them, we perform an OPES simulation where both the s_z and the Deep-LDA CV s_W are biased. We use a deposition rate of 1 ps and a barrier estimate of 50 kJ mol^{-1} for ligands G1, G2, of 60 kJ mol^{-1} for G3 and of 40 kJ mol^{-1} for G4, G5 and G6. Each simulation includes 4 replicas running in parallel, sharing and building together the same bias potential through PLUMED's Multiple Walkers feature. Each replica runs for 140 ns, for a total simulation time of 560 ns.

All the simulation inputs can be found on the PLUMED-NEST repository plumID:20.025. We include below here an example of a PLUMED input for the G4 case.

```
# --- (1) ATOMS DEFINITIONS and ALIGNMENT ---

HOST: GROUP ATOMS=29-224      #host atoms
LIGC: GROUP ATOMS=1-11        #carbon atoms in the ligand
l1: GROUP ATOMS=2             #ligand selected atoms
l2: GROUP ATOMS=3
l3: GROUP ATOMS=11
l4: GROUP ATOMS=14
WO: GROUP ATOMS=234-6533:3    #water oxygen atoms

WHOLEMOLECULES ENTITY0=HOST
FIT_TO_TEMPLATE STRIDE=1 REFERENCE=conf_template.pdb TYPE=OPTIMAL #coordinates alignment
lig: CENTER ATOMS=LIGC

cyl: DISTANCE ATOMS=v1,lig COMPONENTS
```

```
radius: MATHEVAL ARG=cyl.x,cyl.y FUNC=sqrt(x*x+y*y) PERIODIC=NO

v1: FIXEDATOM AT=2.0136,2.0136,2.0   #virtual atoms
v2: FIXEDATOM AT=2.0136,2.0136,2.25
v3: FIXEDATOM AT=2.0136,2.0136,2.5
v4: FIXEDATOM AT=2.0136,2.0136,2.75
v5: FIXEDATOM AT=2.0136,2.0136,3.0
v6: FIXEDATOM AT=2.0136,2.0136,3.25
v7: FIXEDATOM AT=2.0136,2.0136,3.5
v8: FIXEDATOM AT=2.0136,2.0136,3.75

# --- (2) DESCRIPTORS ---

L1: COORDINATION GROUPA=l1 GROUPB=WO SWITCH={RATIONAL D_0=0.0 R_0=0.25 NN=6 MM=10}
NLIST NL_CUTOFF=1.0 NL_STRIDE=5
L2: COORDINATION GROUPA=l2 GROUPB=WO SWITCH={RATIONAL D_0=0.0 R_0=0.25 NN=6 MM=10}
NLIST NL_CUTOFF=1.0 NL_STRIDE=5
L3: COORDINATION GROUPA=l3 GROUPB=WO SWITCH={RATIONAL D_0=0.0 R_0=0.25 NN=6 MM=10}
NLIST NL_CUTOFF=1.0 NL_STRIDE=5
L4: COORDINATION GROUPA=l4 GROUPB=WO SWITCH={RATIONAL D_0=0.0 R_0=0.25 NN=6 MM=10}
NLIST NL_CUTOFF=1.0 NL_STRIDE=5
V1: COORDINATION GROUPA=v1 GROUPB=WO SWITCH={RATIONAL D_0=0.0 R_0=0.25 NN=2 MM=6}
NLIST NL_CUTOFF=1.0 NL_STRIDE=5
V2: COORDINATION GROUPA=v2 GROUPB=WO SWITCH={RATIONAL D_0=0.0 R_0=0.25 NN=2 MM=6}
NLIST NL_CUTOFF=1.0 NL_STRIDE=5
V3: COORDINATION GROUPA=v3 GROUPB=WO SWITCH={RATIONAL D_0=0.0 R_0=0.25 NN=2 MM=6}
NLIST NL_CUTOFF=1.0 NL_STRIDE=5
V4: COORDINATION GROUPA=v4 GROUPB=WO SWITCH={RATIONAL D_0=0.0 R_0=0.25 NN=2 MM=6}
NLIST NL_CUTOFF=1.0 NL_STRIDE=5
V5: COORDINATION GROUPA=v5 GROUPB=WO SWITCH={RATIONAL D_0=0.0 R_0=0.25 NN=2 MM=6}
NLIST NL_CUTOFF=1.0 NL_STRIDE=5
V6: COORDINATION GROUPA=v6 GROUPB=WO SWITCH={RATIONAL D_0=0.0 R_0=0.25 NN=2 MM=6}
NLIST NL_CUTOFF=1.0 NL_STRIDE=5
V7: COORDINATION GROUPA=v7 GROUPB=WO SWITCH={RATIONAL D_0=0.0 R_0=0.25 NN=2 MM=6}
NLIST NL_CUTOFF=1.0 NL_STRIDE=5
V8: COORDINATION GROUPA=v8 GROUPB=WO SWITCH={RATIONAL D_0=0.0 R_0=0.25 NN=2 MM=6}
NLIST NL_CUTOFF=1.0 NL_STRIDE=5

d1: MATHEVAL ARG=L1 FUNC=(x/2.5)-1.0 PERIODIC=NO  #normalized descriptors
d2: MATHEVAL ARG=L2 FUNC=(x/2.5)-1.0 PERIODIC=NO
d3: MATHEVAL ARG=L3 FUNC=(x/2.5)-1.0 PERIODIC=NO
d4: MATHEVAL ARG=L4 FUNC=(x/2.5)-1.0 PERIODIC=NO
d5: MATHEVAL ARG=V1 FUNC=(x/2.8)-1.0 PERIODIC=NO
d6: MATHEVAL ARG=V2 FUNC=(x/2.8)-1.0 PERIODIC=NO
d7: MATHEVAL ARG=V3 FUNC=(x/2.8)-1.0 PERIODIC=NO
d8: MATHEVAL ARG=V4 FUNC=(x/2.8)-1.0 PERIODIC=NO
d9: MATHEVAL ARG=V5 FUNC=(x/2.8)-1.0 PERIODIC=NO
d10: MATHEVAL ARG=V6 FUNC=(x/2.8)-1.0 PERIODIC=NO
d11: MATHEVAL ARG=V7 FUNC=(x/2.8)-1.0 PERIODIC=NO
d12: MATHEVAL ARG=V8 FUNC=(x/2.8)-1.0 PERIODIC=NO
```

```
# --- (3) DEEP-LDA CV and other quantities ---

s: PYTORCH_MODEL MODEL=modelG4_a.pt ARG=d1,d2,d3,d4,d5,d6,d7,d8,d9,d10,d11,d12  #NN output
sw: MATHEVAL ARG=s.node-0 FUNC=x+x^3 PERIODIC=NO   #Deep-LDA CV

funnel: MATHEVAL ARG=radius,cyl.z VAR=r,z FUNC=(r+1.0*(-1.2+z))*step(-z+1.)+(r-0.2)*step(z-1.)
PERIODIC=NO
UPPER_WALLS AT=0 ARG=funnel KAPPA=2000.0 LABEL=funnelwall  #funnel restraint
UPPER_WALLS AT=1.8 ARG=cyl.z KAPPA=4000.0 EXP=2 LABEL=sz_wall  #upper limit of s_z

ang: ANGLE ATOMS=v3,v5,6,11   #angle of a ligand's axis with z
cosang: MATHEVAL ARG=ang FUNC=cos(x) PERIODIC=NO

# --- (4) OPES  ---

OPES_METAD ...
   LABEL=opes
   ARG=cyl.z,sw
   FILE=Kernels.data
   STATE_WFILE=compressed_Kernels.data
   PACE=500
   BARRIER=40
   WALKERS_MPI
... OPES_METAD

PRINT ARG=* STRIDE=250 FILE=COLVAR FMT=%8.4f

ENDPLUMED
```

Calculating average properties

For calculating average properties, such as binding free energies and estimating their statistical error, we apply the following strategy. Given a guest and a Deep-LDA CV, for each simulation we invert the trajectories of each of the 4 replicas and merge them into one longer trajectory. This way, the last configurations of each replica at the last timestesp 140 ns become the first 4 configurations in the new trajectory and so on. This step helps when calculating free energies, as it simplifies the discarding of the initial non-equilibrated part of the simulations.

Then, for numerical stability, we filter out the outlier configurations where the funnel restraint has a bias energy larger than 10 kJ mol^{-1}. We split the resulting trajectory into 5 blocks of 100 ns each, where block 1 corresponds to the last part of the simulations and block 5 to the initial one. The very beginning of the simulations is not taken into account in a natural way. In each block b, we independently evaluate the FES and the binding free energy ΔG_b through Eq. 4. Every block has a different statistical weight given by $w_\mathrm{b} = \sum_j e^{\beta V(j)}$ where index j runs over the configurations in the block and V is the bias from OPES.

The mean ΔG is then simply the weighted average over the blocks

$$\Delta G = \frac{\sum_{b=1}^{5} w_b \, \Delta G_\mathrm{b}}{\sum_{b=1}^{5} w_b} \tag{5}$$

and its error the weighted standard deviation

$$\sigma(\Delta G) = \sqrt{\frac{1}{N_\mathrm{eff} - 1} \, \frac{\sum_{b=1}^{5} w_b \, (\Delta G_\mathrm{b} - \Delta G)^2}{\sum_{b=1}^{5} w_b}}. \tag{6}$$

The effective sample size

$$N_{\text{eff}} = \frac{\left(\sum_{b=1}^{5} w_b\right)^2}{\sum_{b=1}^{5} w_b^2} \tag{7}$$

measures the level of correlations between the blocks. In the ideal case of low correlation, $N_{\text{eff}} \to 5$, i.e. the number of blocks. Our calculations present a reasonably low level of correlations between blocks as the lowest value that we observed is $N_{\text{eff}} \approx 4.5$.

In the results breakdown below, for each guest molecule and Deep-LDA CV we show in tables ΔG_{b}, w_{b} and the average ΔG with its error. In the main text, we also present in Tab. 1 the average binding energy for every ligand where we applied the weighted averaging procedure to all the blocks of all the CVs.

Supplementary Figures

Host OAMe

Water behaviour in the ligand-free state

We first present a study of the interaction of the host with the water in absence of a guest molecule. The simulations are static bias molecular dynamics simulations of about 80 ns. The static bias was generated from a preceding OPES simulation where CV V_2 was biased with a barrier of 30 kJ mol^{-1} for about 80 ns.

In order to calculate the number of water molecules inside the host $P(n)$ in Fig 6 (b) in the main text, we create an alpha-shape using the backbone carbon atoms coordinate of the host molecule (see Supplementary Fig. 1). The volume enclosed in the alpha-shape makes it a convenient to define whether a point (here, every water oxygen atom) lies inside or outside of the host binding pocket.

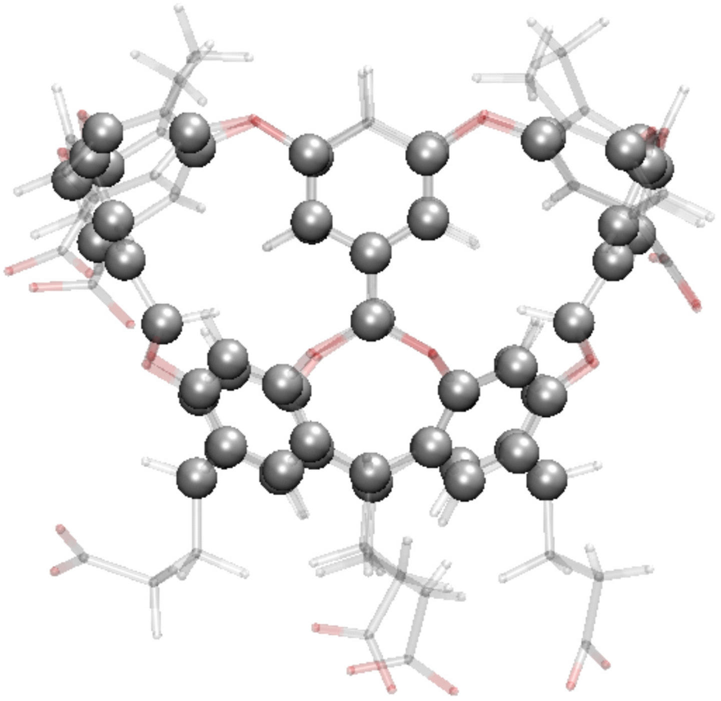

Supplementary Figure 1: **Alpha-shape definition.** The grey spheres indicate the carbon atoms of the host molecule that we use to construct an alpha-shape.

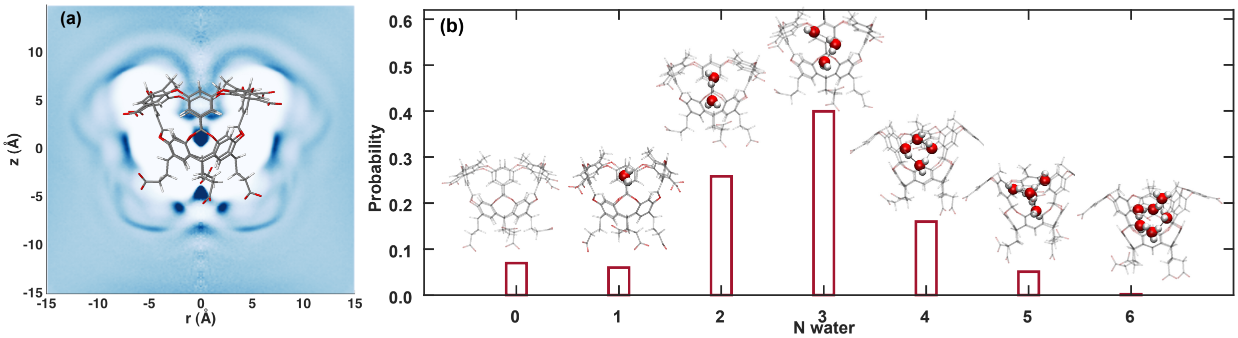

Supplementary Figure 2: **Water distribution analysis in the presence of the OAMe host without a guest.** To better understand how water interacts with the host, we simulate the host OAMe in the absence of a ligand. We run an OPES simulation where we enhance the motion of the water in and out of the pocket by depositing bias on descriptor V_2 and recover unbiased information through standard reweighting. In (a), histogram in cylindrical coordinates z, r representing the presence of the water oxygen atoms around the host without any guest molecule being present. Darker colours correspond to a higher water density. We observe that there is a high probability of finding a water molecule at the centre of the cavity in proximity of the 8 equatorial oxygen atoms. An analysis of the charge distribution shows that this position is a minimum of the electrostatic potential (see Supplementary Fig. 4). Starting from this position a short wire of hydrogen-bonded water molecules can form inside the cavity. As indicated by the density bands, this wire can possibly link up with water outside the pocket. In (b) we report the probability distribution of the number of water molecules inside the pocket in the absence of a guest molecule. We show the snapshots of typical configurations for each case. We find that the number of water molecules inside the cavity exhibits a broad distribution of states. A typical wet configuration is the one in which three water molecules form a linear cluster inside the cavity, in agreement with results in an analogous system [17].

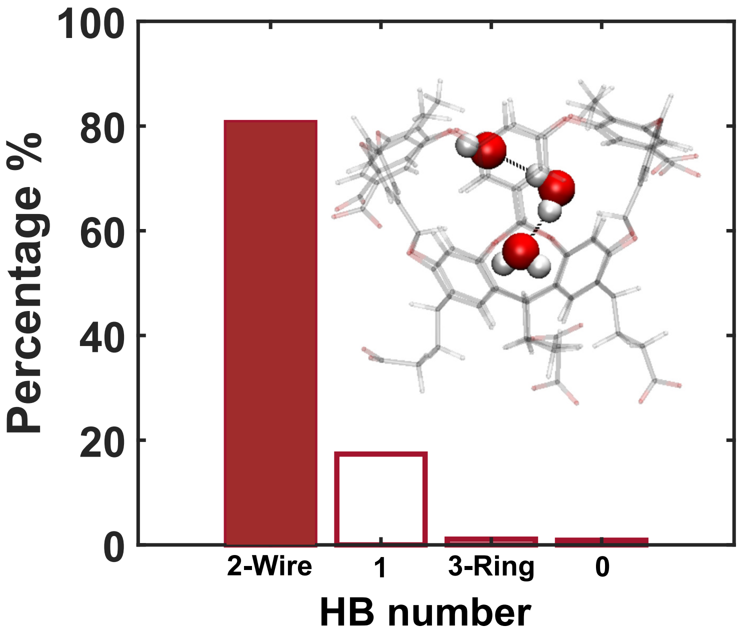

Supplementary Figure 3: **Probability distribution of the number of hydrogen bonds between water molecules in the case where 3 water molecules are inside the pocket.** In the inset, we show one representative snapshot of the most probable case where cavity presents three water molecules in a wire configuration.

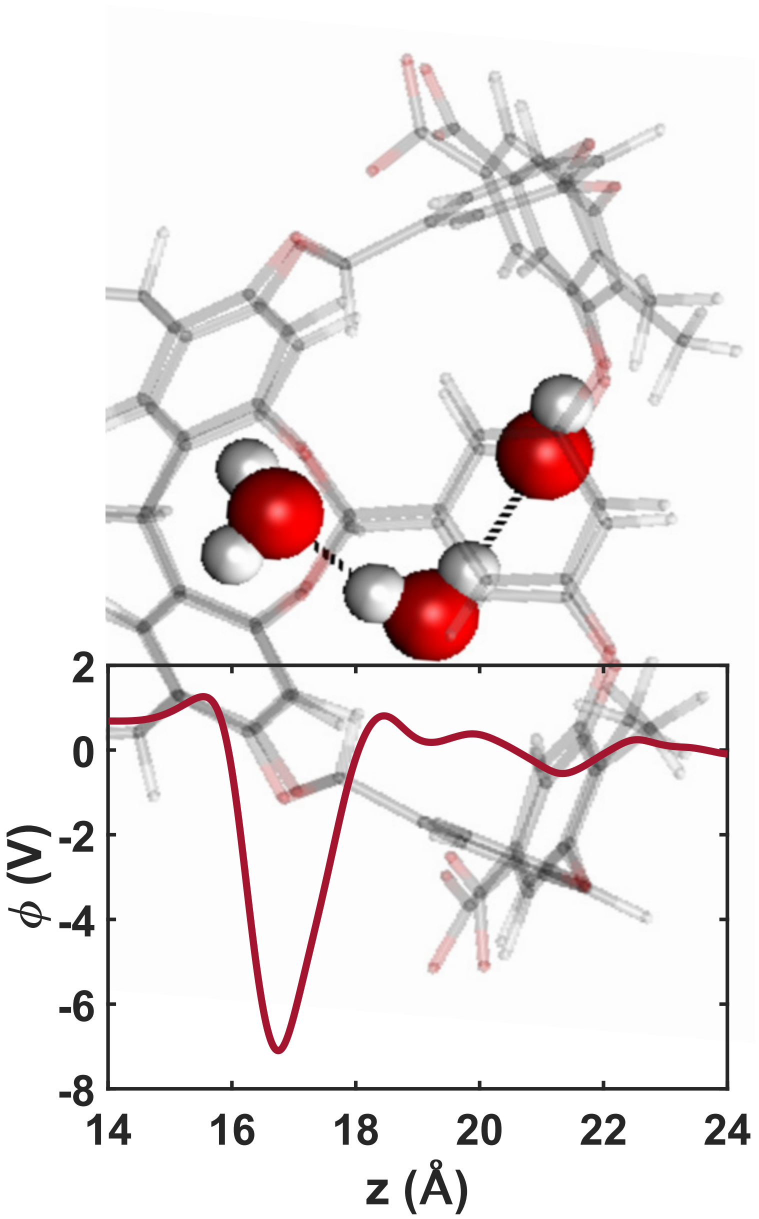

Supplementary Figure 4: **Electrostatic potential along the binding axis.** We also show the corresponding snapshot of a typical configuration with 3 water molecule aligned in the pocket.

Ligand Binding

We analyse the simulations between host OAMe and each ligand. The results are presented in Supplementary Fig. 5-30 and Tab. 1-8.

At first, we show a trajectory comparison. We perform identical simulations with respect to the Deep-LDA ones, with the only difference that instead of biasing a Deep-LDA CV we bias another commonly used physical variable, $\cos(\theta)$, where θ is the angle between the z axis and a ligand axis. We show the dynamics of the 4 replicas in this case and compare with the corresponding Deep-LDA simulations. Visual inspection of the trajectories indicates that in most cases the use of the Deep-LDA CV brings about a faster transition rate that leads to an improved phase space exploration.

The trajectories are coloured with the instantaneous value of the Deep-LDA CV and we notice a clear difference between the two. The simulations with CV $\cos(\theta)$ stay for long stretches of time in a constant colour area, which corresponds to the system staying in the same state, not performing any transition. On the other hand, the Deep-LDA simulations tend to have a dynamics that rapidly covers the whole range of colours which indicates a thorough phase space exploration.

Then, we present Figures analogous to Fig. 5 in the main text, where we plot a 2-dimensional FES over s_z and s_w and analyse the water position in different states. At last, we present a 2-dimensional FES over s_z and the coordination number around virtual atom V_2 and highlight the different states.

To assess the quality of our results, we perform the error analysis proposed in [1] and repeat the procedure that the authors made available in the public repository https://github.com/GilsonLabUCSD/SAMPL5-bootstrapping-error-analysis performing 10^5 bootstrap cycles. The resulting error statistics is shown in Supplementary Tab. 2. We compare the results of our method Deep-LDA with methods using the same model: Metadynamics [4], the attach-pull-release method (APR) [2] and an alchemical method (SOMD-3) [3]. When available, we used the latest results that the authors provided in the papers, after the SAMPL5 official submission.

OAMe-G1

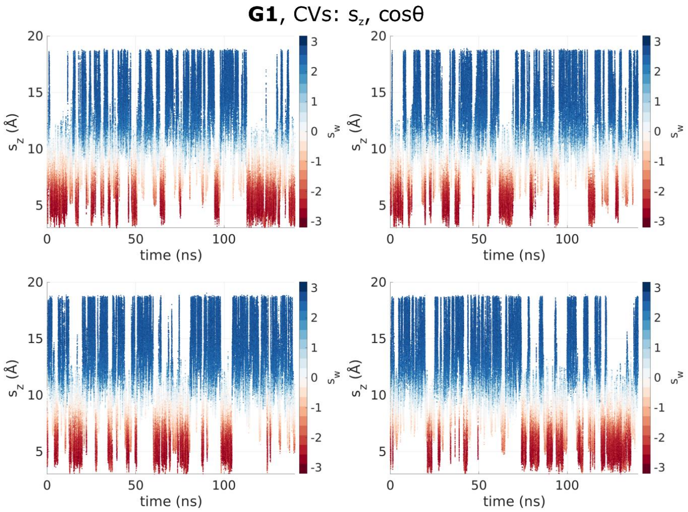

Supplementary Figure 5: **Standard OAMe-G1 trajectories.** We show the dynamics of s_z in an OPES simulation where s_z and $\cos\theta$ are biased. The plot is coloured with the instantaneous value of s_w.

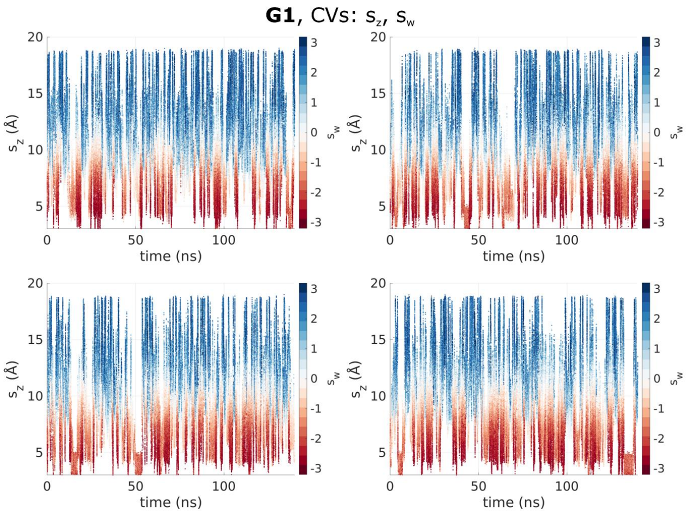

Supplementary Figure 6: **Deep-LDA OAMe-G1 trajectories.** We show the dynamics of s_z in an OPES simulation where s_z and s_w are biased. The plot is coloured with the instantaneous value of s_w.

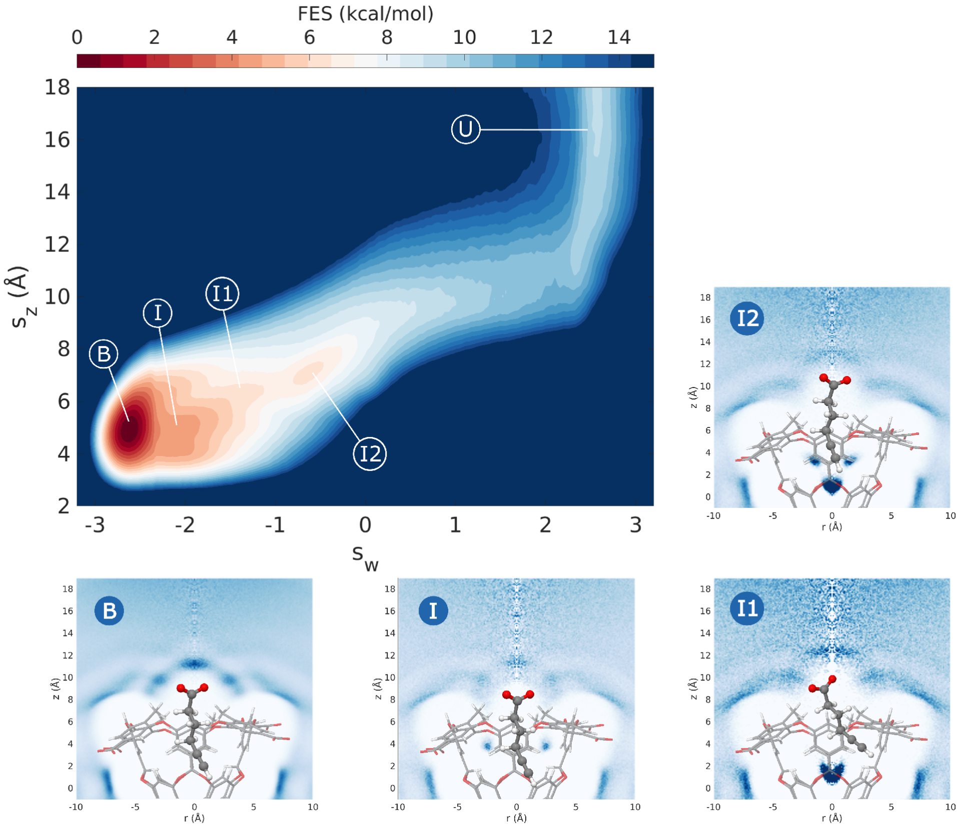

Supplementary Figure 7: **OAMe-G1 FES with respect to** s_z **and** s_w. We also select some relevant states and perform unbiased simulations to measure the presence of water. We show histograms of the water oxygen atoms density in cylindrical coordinates z, r in these states with an illustrative sketch of the guest and the host. Darker colours correspond to a higher water density.

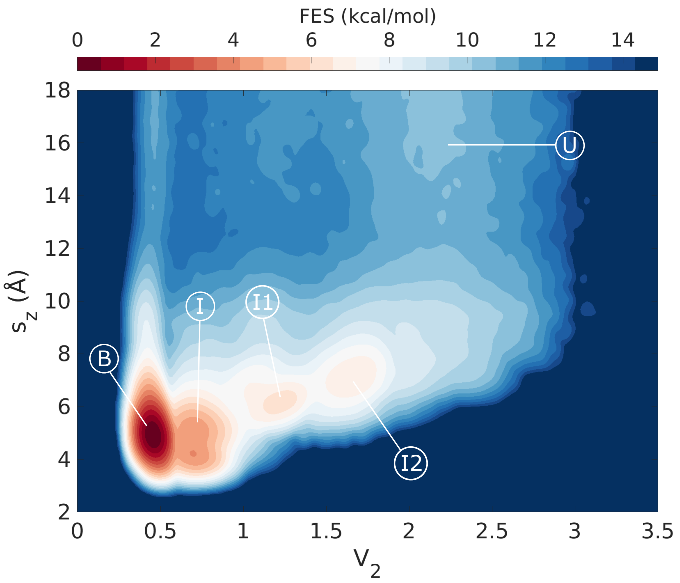

Supplementary Figure 8: **OAMe-G1 FES with respect to** s_z **and** V_2**.** V_2 here measures the water presence in the cavity and is a non-normalized coordination number.

OAMe-G2

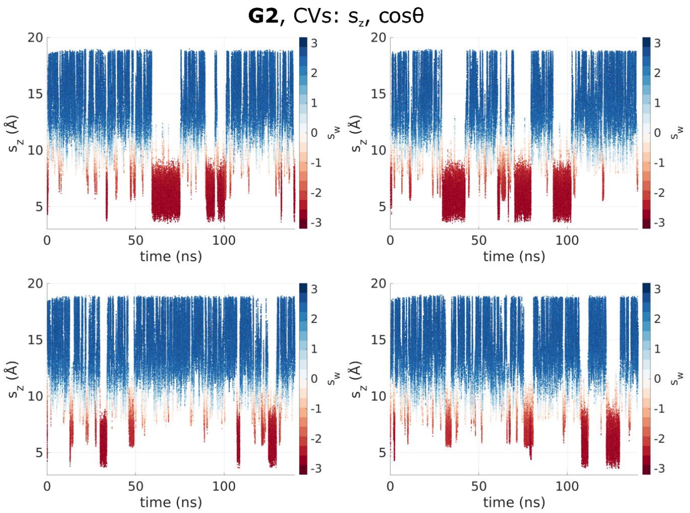

Supplementary Figure 9: **Standard OAMe-G2 trajectories.** We show the dynamics of s_z in an OPES simulation where s_z and $\cos\theta$ are biased. The plot is coloured with the instantaneous value of s_w.

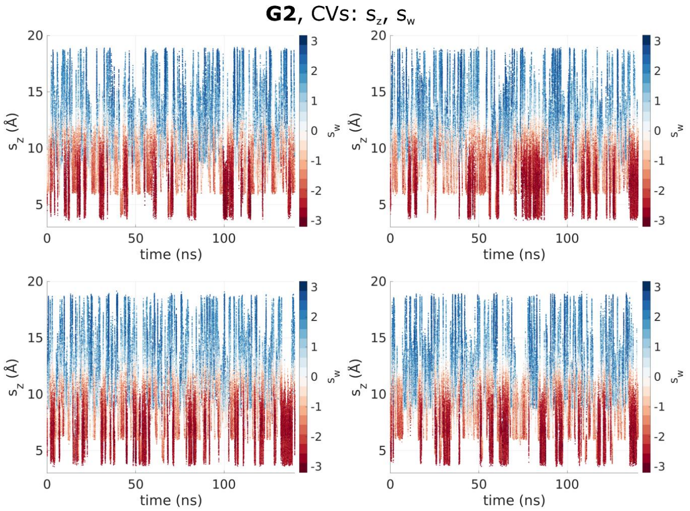

Supplementary Figure 10: **Deep-LDA OAMe-G2 trajectories.** We show the dynamics of s_z in an OPES simulation where s_z and s_w are biased. The plot is coloured with the instantaneous value of s_w.

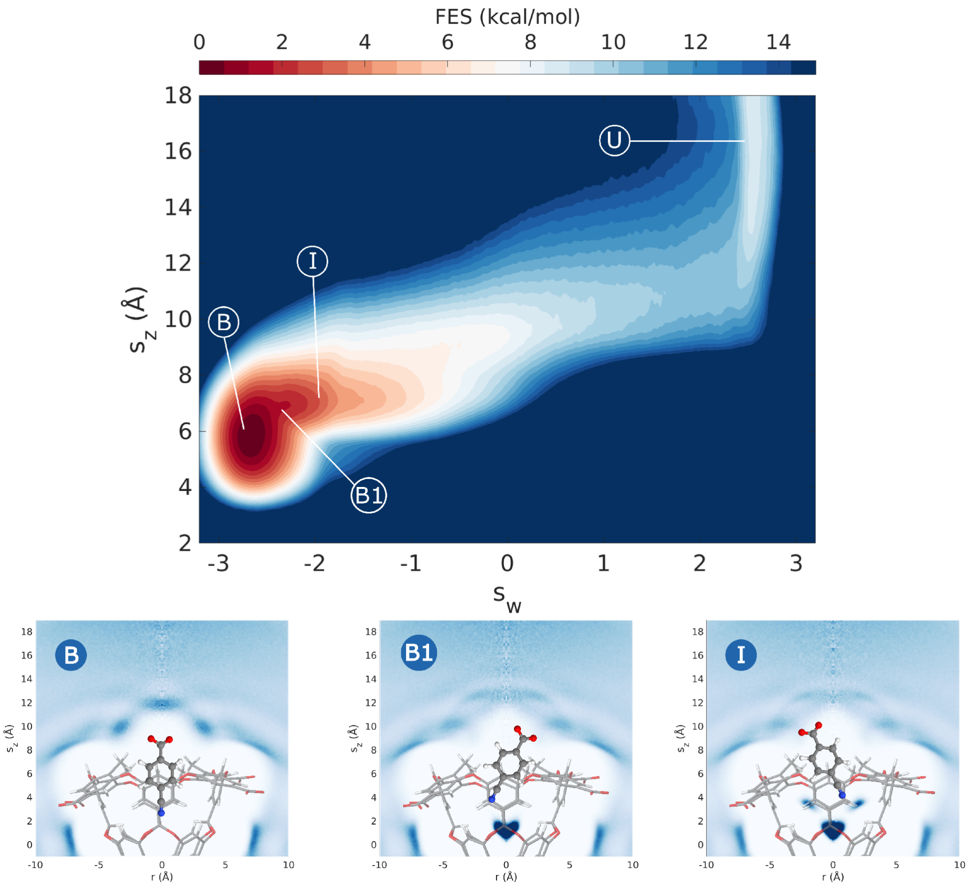

Supplementary Figure 11: **OAMe-G2 FES with respect to s_z and s_w.**

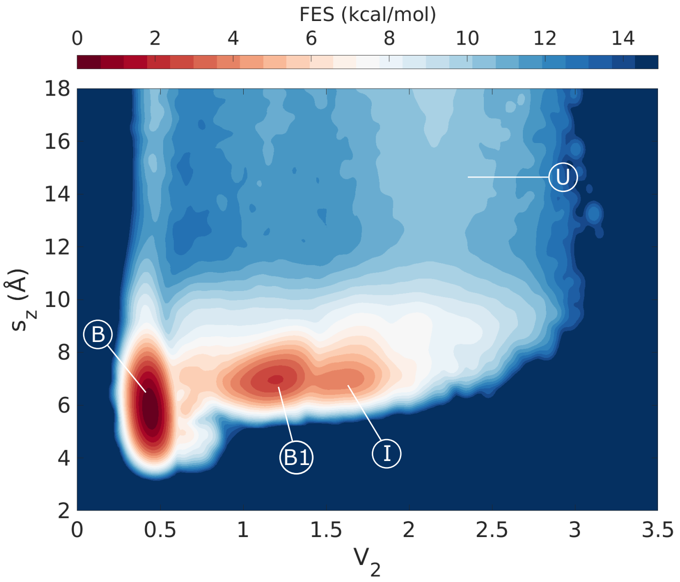

Supplementary Figure 12: **OAMe-G2 FES with respect to s_z and V_2.**

OAMe-G3

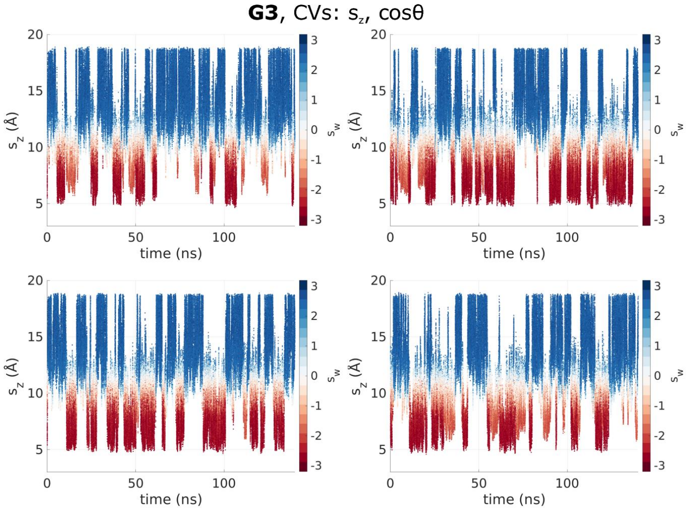

Supplementary Figure 13: **Standard OAMe-G3 trajectories.** We show the dynamics of s_z in an OPES simulation where s_z and $\cos\theta$ are biased. The plot is coloured with the instantaneous value of s_w.

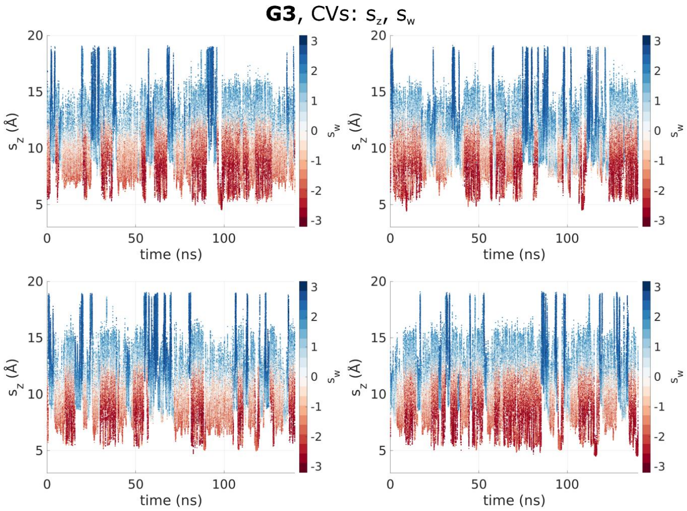

Supplementary Figure 14: **Deep-LDA OAMe-G3 trajectories.** We show the dynamics of s_z in an OPES simulation where s_z and s_w are biased. The plot is coloured with the instantaneous value of s_w.

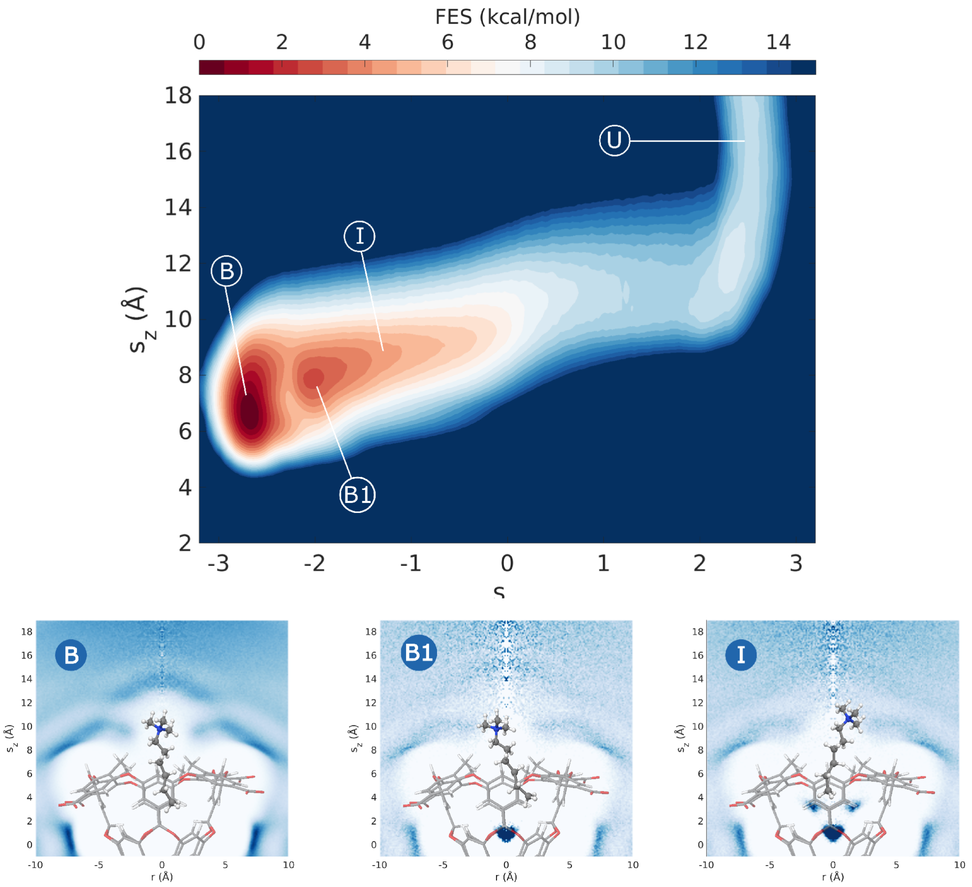

Supplementary Figure 15: **OAMe-G3 FES with respect to s_z and s_w.**

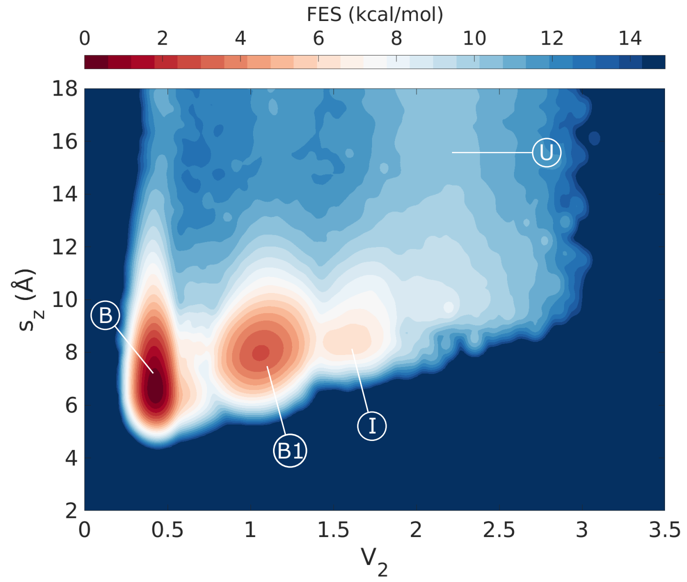

Supplementary Figure 16: **OAMe-G3 FES with respect to s_z and V_2.**

OAMe-G4

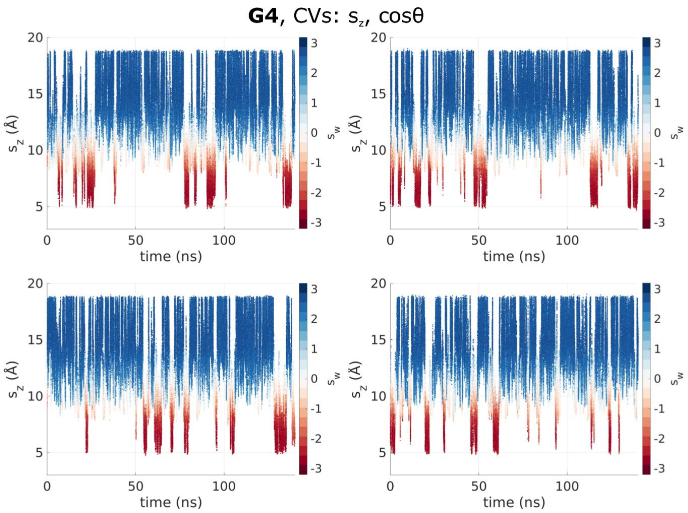

Supplementary Figure 17: **Standard OAMe-G4 trajectories.** We show the dynamics of s_z in an OPES simulation where s_z and $\cos\theta$ are biased. The plot is coloured with the instantaneous value of s_w.

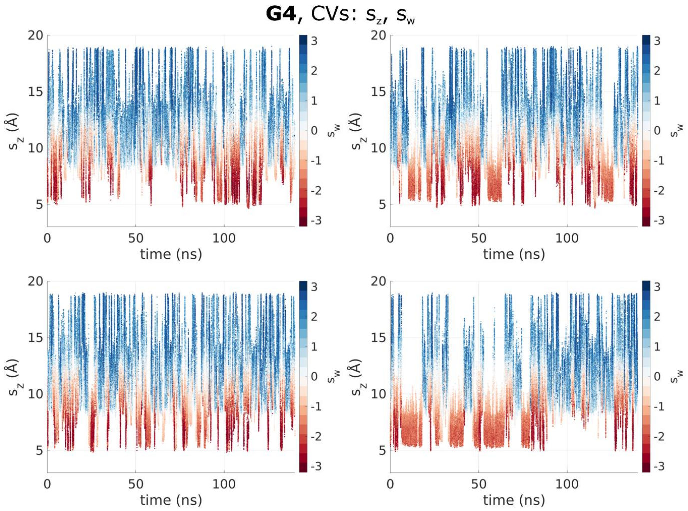

Supplementary Figure 18: **Deep-LDA OAMe-G4 trajectories.** We show the dynamics of s_z in an OPES simulation where s_z and s_w are biased. The plot is coloured with the instantaneous value of s_w.

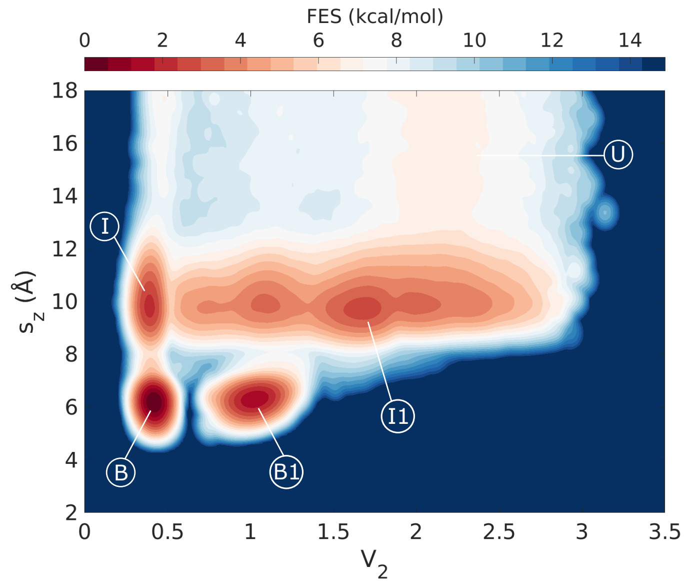

Supplementary Figure 19: **OAMe-G4 FES with respect to s_z and V_2.**

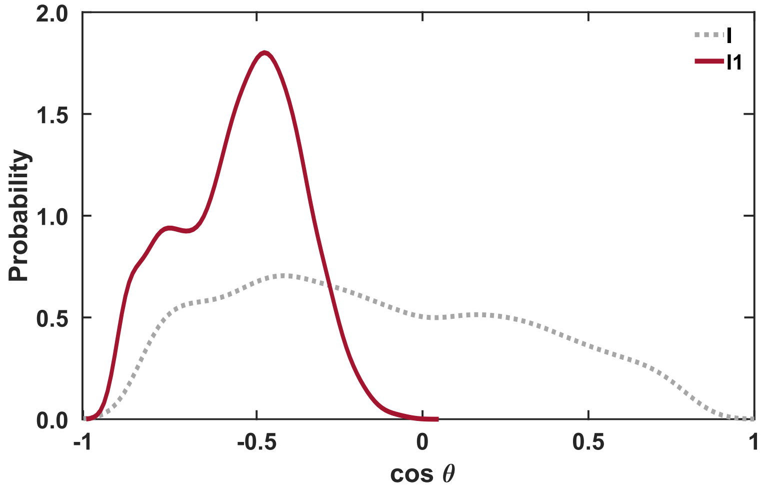

Supplementary Figure 20: **Ligand angular distribution in OAMe-G4 intermediate states.** Probability distribution of $\cos\theta$ for the G4 molecule in the intermediate states I and I1, where θ is the angle between the binding axis and the -COO group in the ligand.

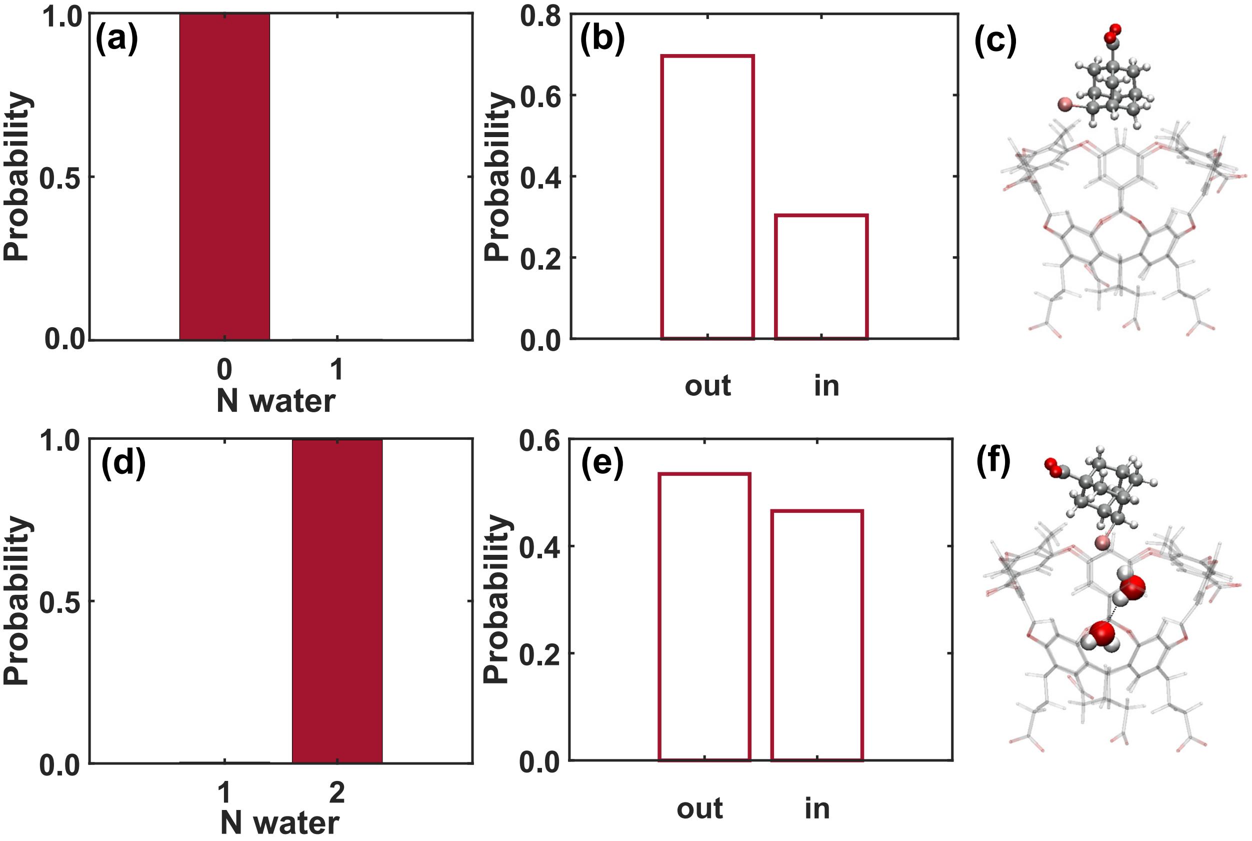

Supplementary Figure 21: **Water analysis of the OAMe-G4 intermediate states.** In (a) and (d), probability distributions of the number of water molecules within the pocket for respectively intermediate states I and I1. In (b) and (e), probability distribution of the Br atom orientation with respect to the pocket for respectively intermediate states I and I1. In (c), a snapshot of the dry pocket with the "out" configuration for the Br atom in state I. In (f), a snapshot of the wet pocket with 2 water molecules with the "in" configuration for the Br atom in state I1.

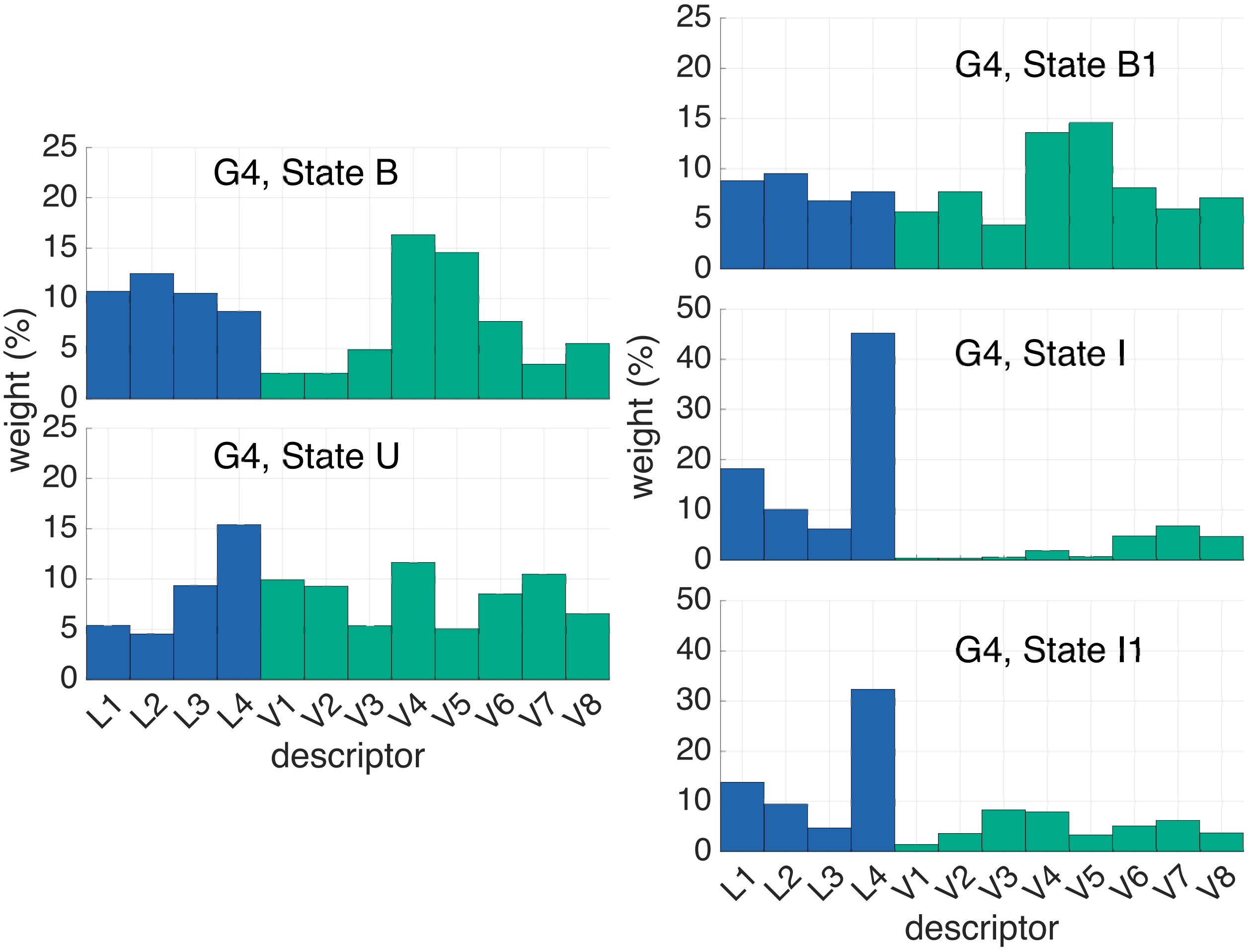

Supplementary Figure 22: **Descriptors relative weights for OAMe-G4.** Same as Fig. 6 in the main text, for all the metastable states of OAMe-G4.

OAMe-G5

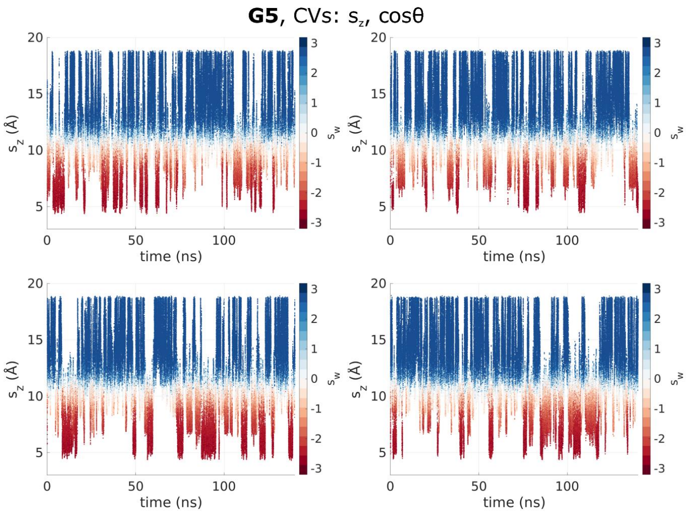

Supplementary Figure 23: **Standard OAMe-G5 trajectories.** We show the dynamics of s_z in an OPES simulation where s_z and $\cos\theta$ are biased. The plot is coloured with the instantaneous value of s_w.

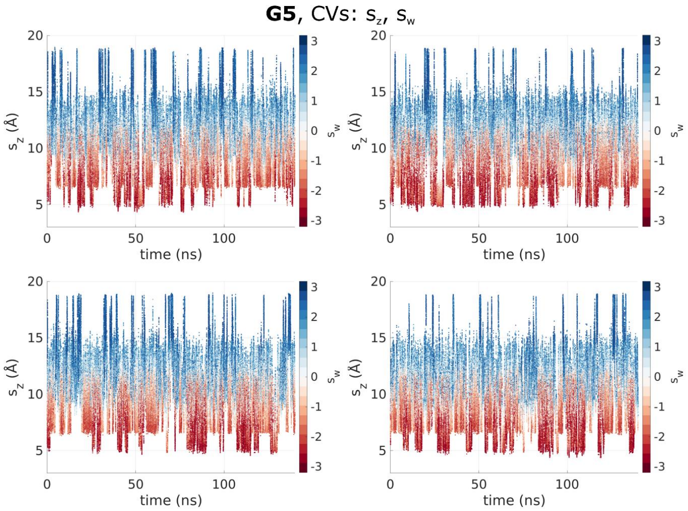

Supplementary Figure 24: **Deep-LDA OAMe-G5 trajectories.** We show the dynamics of s_z in an OPES simulation where s_z and s_w are biased. The plot is coloured with the instantaneous value of s_w.

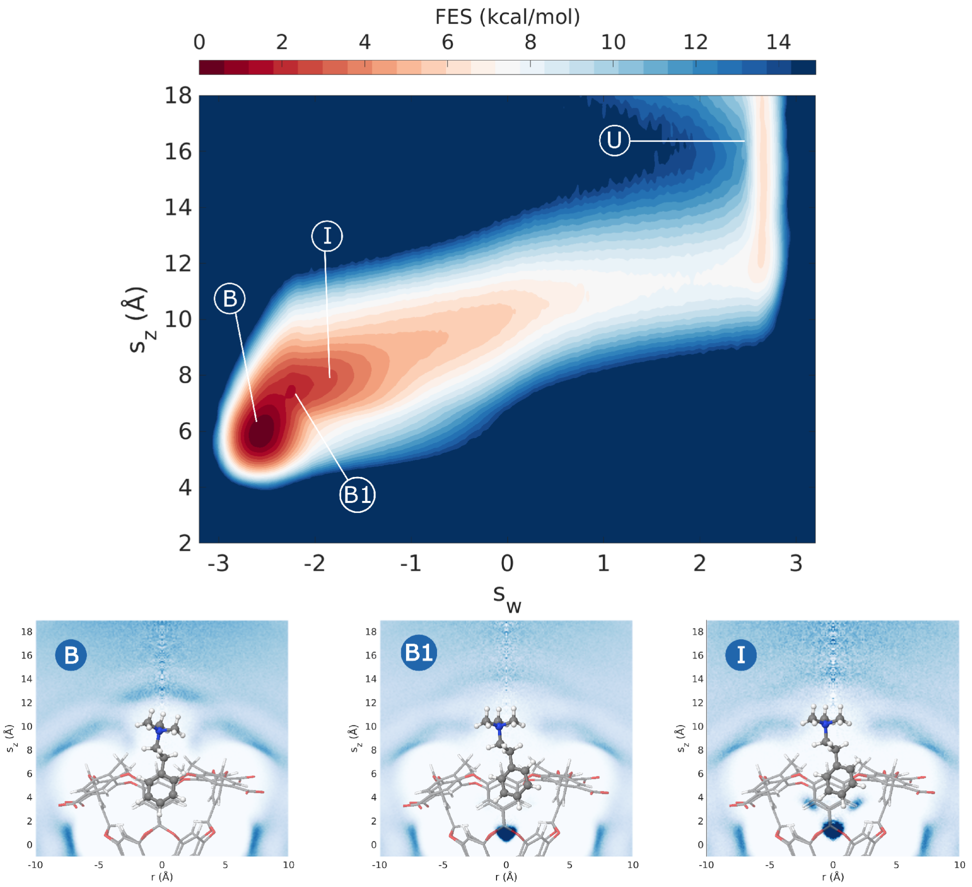

Supplementary Figure 25: **OAMe-G5 FES with respect to s_z and s_w.**

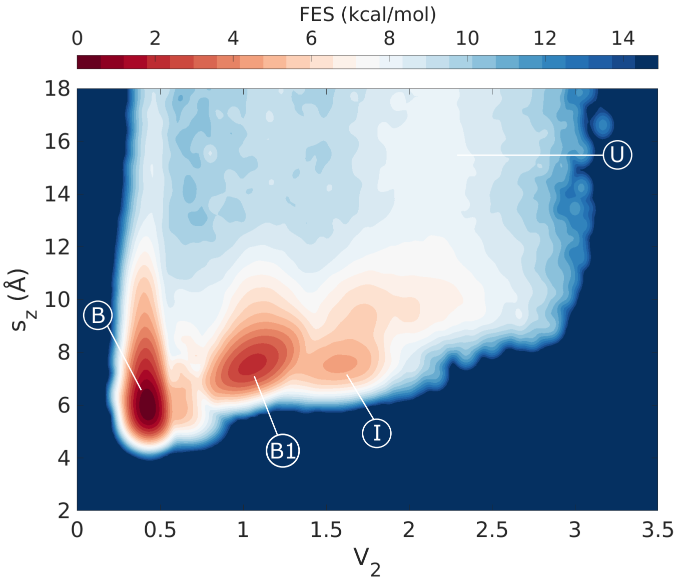

Supplementary Figure 26: **OAMe-G5 FES with respect to s_z and V_2.**

OAMe-G6

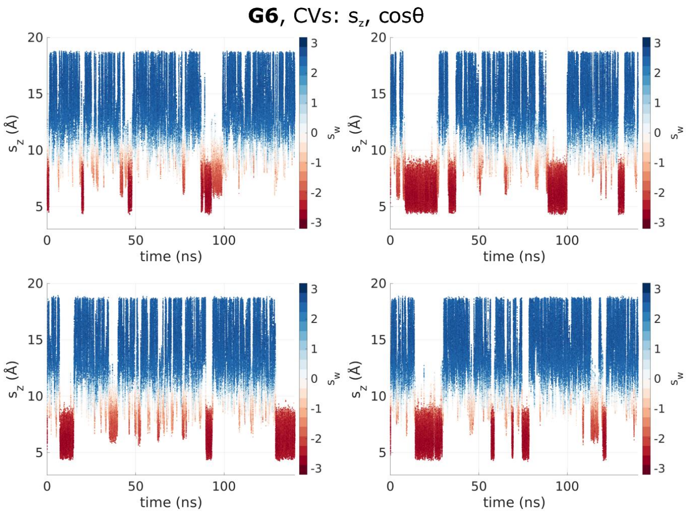

Supplementary Figure 27: **Standard OAMe-G6 trajectories.** We show the dynamics of s_z in an OPES simulation where s_z and $\cos\theta$ are biased. The plot is coloured with the instantaneous value of s_w.

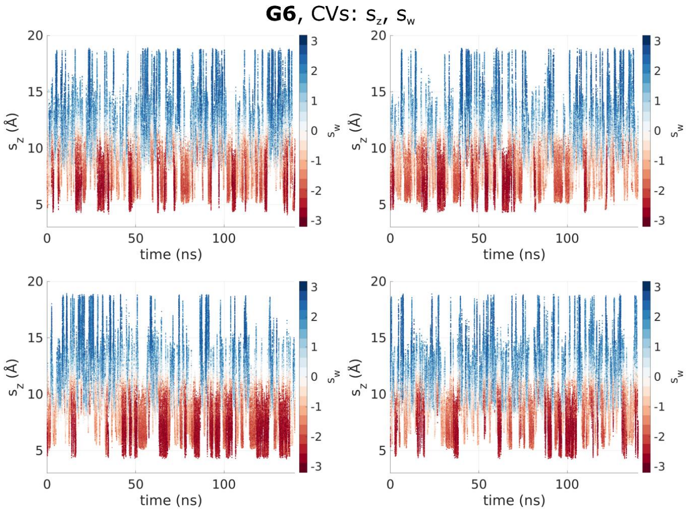

Supplementary Figure 28: **Deep-LDA OAMe-G6 trajectories.** We show the dynamics of s_z in an OPES simulation where s_z and s_w are biased. The plot is coloured with the instantaneous value of s_w.

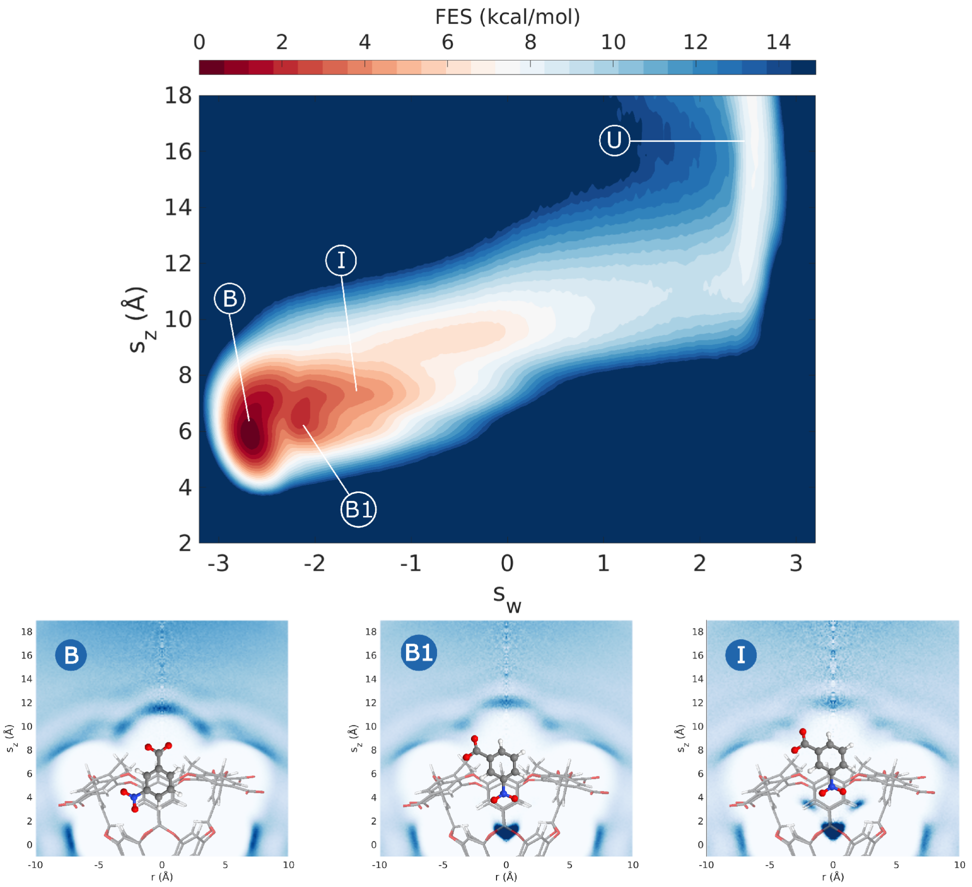

Supplementary Figure 29: **OAMe-G6 FES with respect to** s_z **and** s_w.

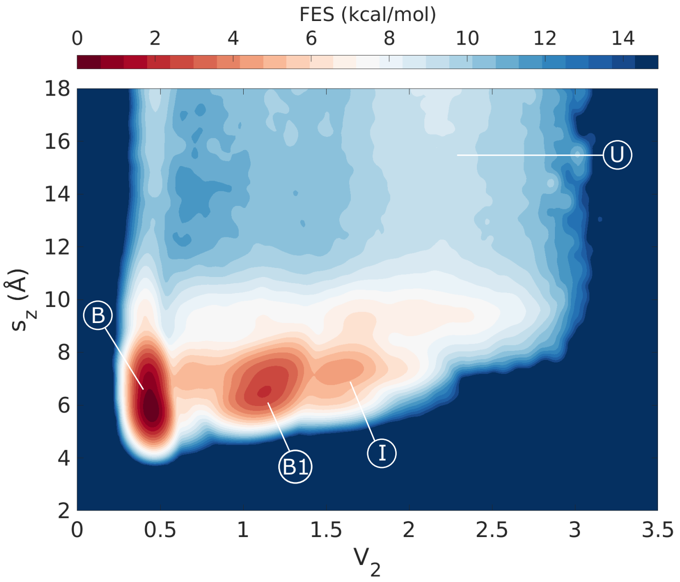

Supplementary Figure 30: **OAMe-G6 FES with respect to** s_z **and** V_2**.**

Host OAH

To highlight the robustness of the Deep-LDA procedure, we present here a set of binding results between another host from the SAMPL5 challenge, namely OAH, and the same six guest molecules used previoulsy. The procedure is completely analogous to the one used for host OAMe. In the OPES simulations, we used a barrier estimate of 50 kJ mol^{-1} for all ligands except for G4 where we used 90 kJ mol^{-1}. The results are in line with the previous system and can be found in Supplementary Fig. 31-57 and Supplementary Tab. 9-16.

Water behaviour in the ligand-free state

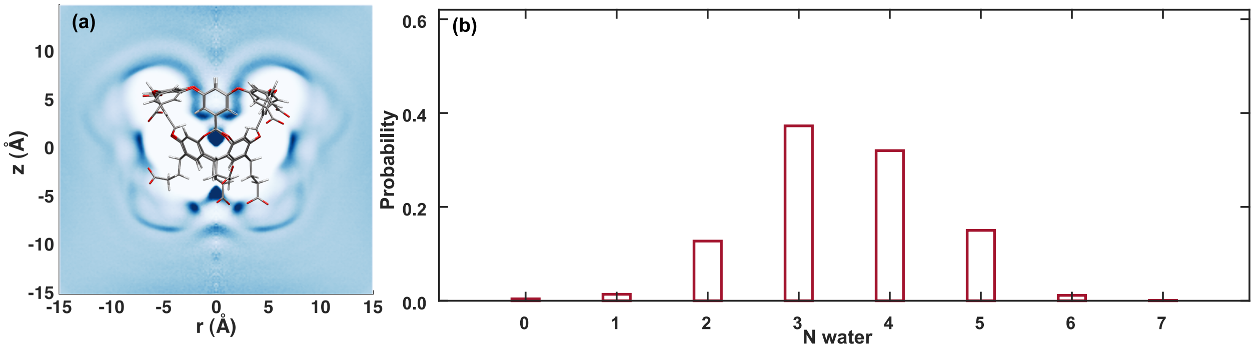

Supplementary Figure 31: **Water distribution analysis in the presence of the OAH host without a guest.** Same as Supplementary Fig. 2 for host OAH.

Ligand Binding

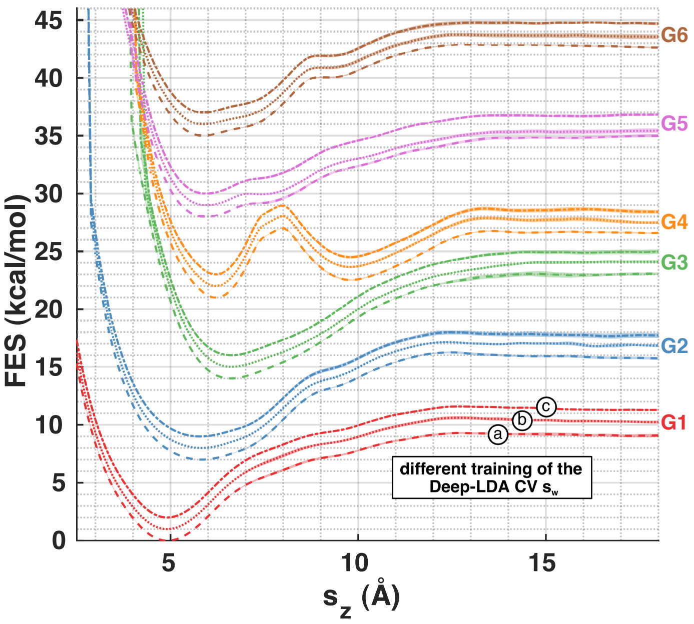

Supplementary Figure 32: **Free energy surfaces projected along the host-guest distance.** Same as Supplementary Fig. 3 for host OAH.

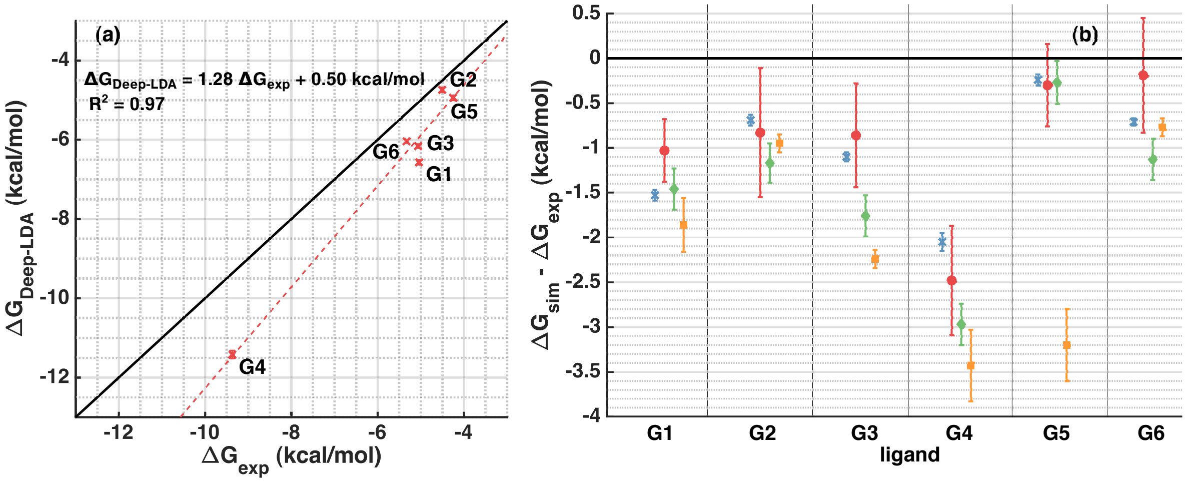

Supplementary Figure 33: **Comparison of the binding free energies with experiments and other calculations.** Same as Supplementary Fig. 4 for host OAH.

OAH-G1

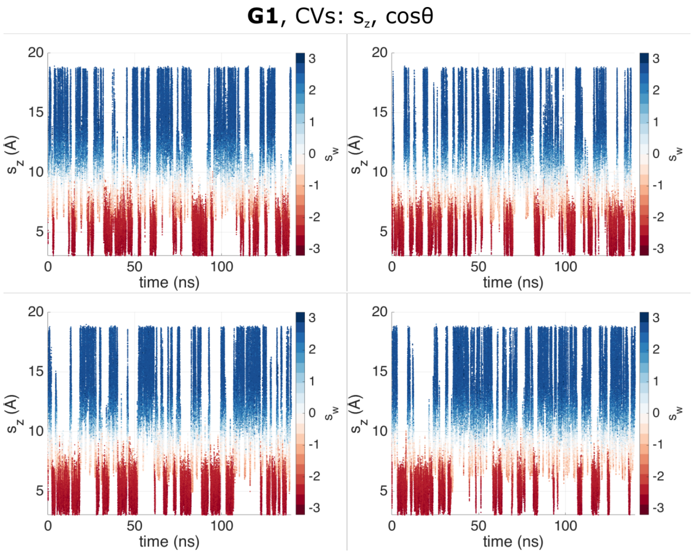

Supplementary Figure 34: **Standard OAH-G1 trajectories.** We show the dynamics of s_z in an OPES simulation where s_z and $\cos\theta$ are biased. The plot is coloured with the instantaneous value of s_{w}.

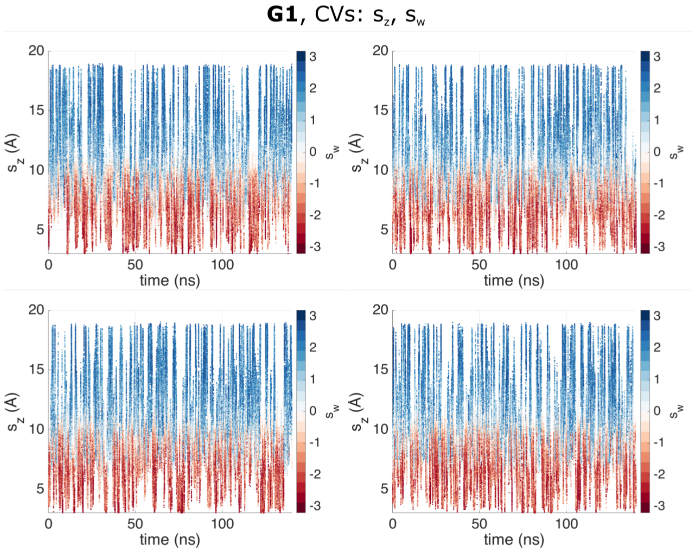

Supplementary Figure 35: **Deep-LDA OAH-G1 trajectories.** We show the dynamics of s_z in an OPES simulation where s_z and s_w are biased. The plot is coloured with the instantaneous value of s_w.

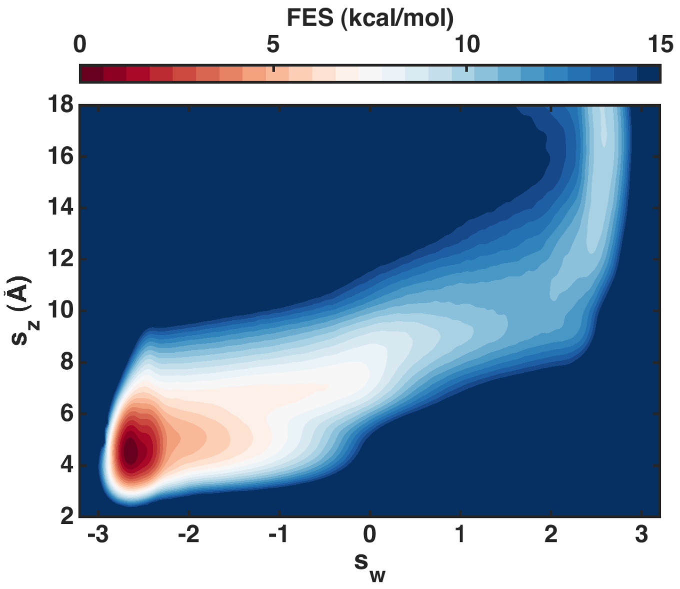

Supplementary Figure 36: **OAH-G1 FES with respect to s_z and s_w.**

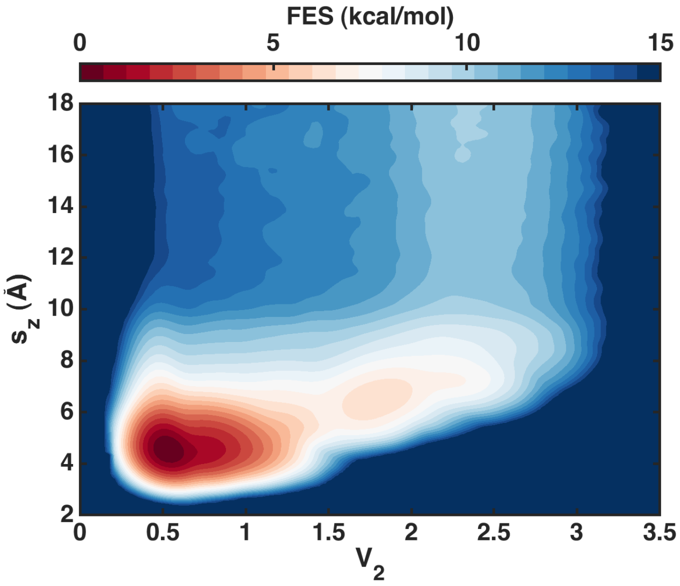

Supplementary Figure 37: **OAH-G1 FES with respect to s_z and V_2.**

OAH-G2

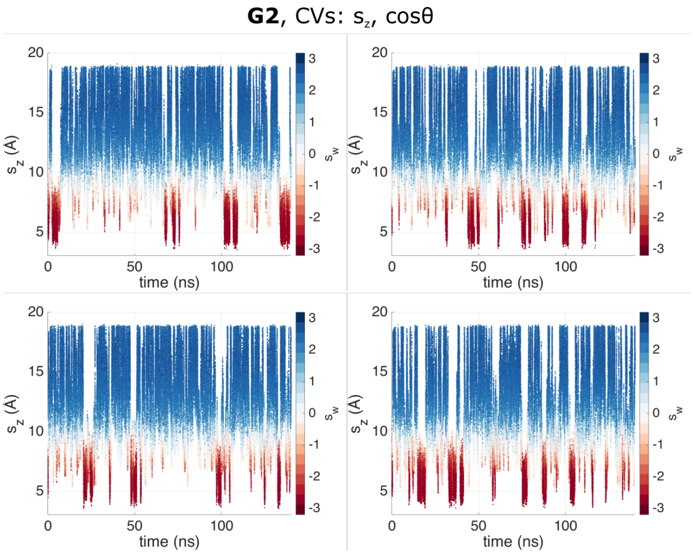

Supplementary Figure 38: **Standard OAH-G2 trajectories.** We show the dynamics of s_z in an OPES simulation where s_z and $\cos\theta$ are biased. The plot is coloured with the instantaneous value of s_{w}.

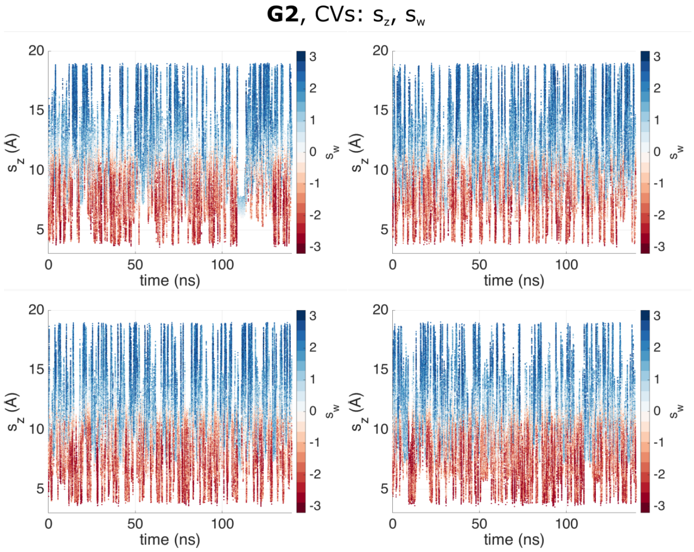

Supplementary Figure 39: **Deep-LDA OAH-G2 trajectories.** We show the dynamics of s_z in an OPES simulation where s_z and s_w are biased. The plot is coloured with the instantaneous value of s_w.

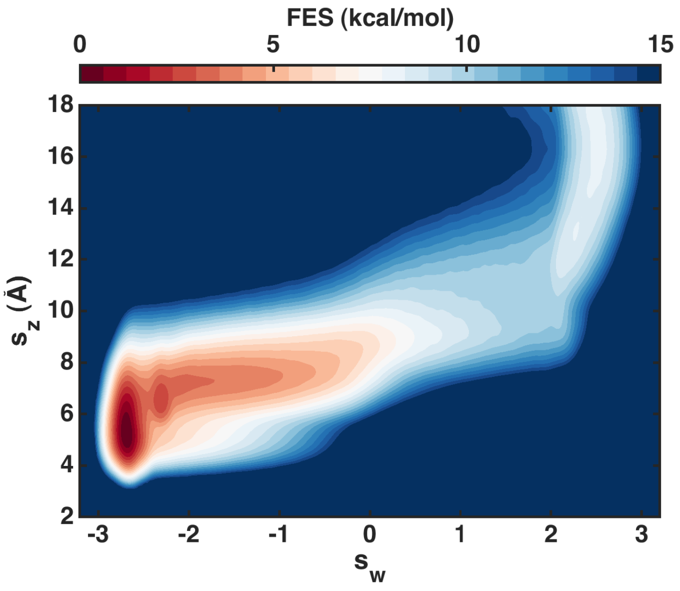

Supplementary Figure 40: **OAH-G2 FES with respect to s_z and s_w.**

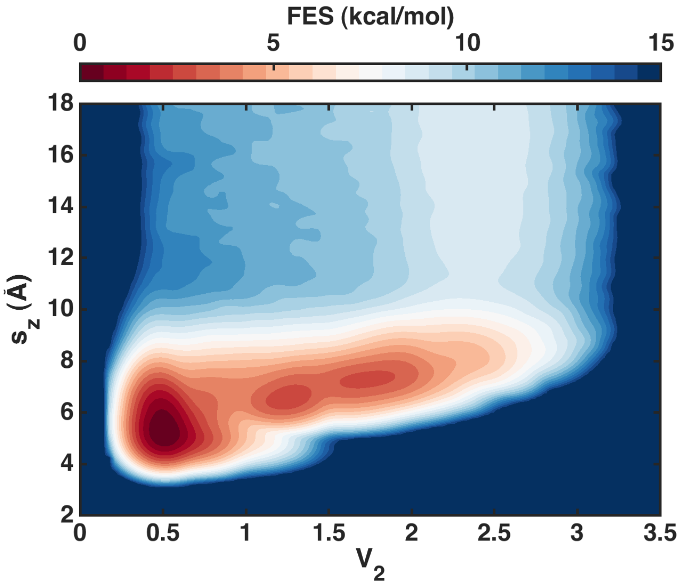

Supplementary Figure 41: **OAH-G2 FES with respect to s_z and V_2.**

OAH-G3

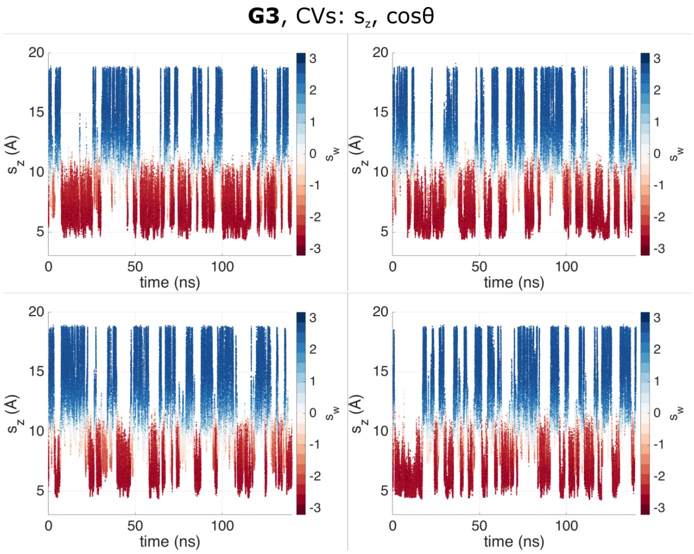

Supplementary Figure 42: **Standard OAH-G3 trajectories.** We show the dynamics of s_z in an OPES simulation where s_z and $\cos\theta$ are biased. The plot is coloured with the instantaneous value of s_w.

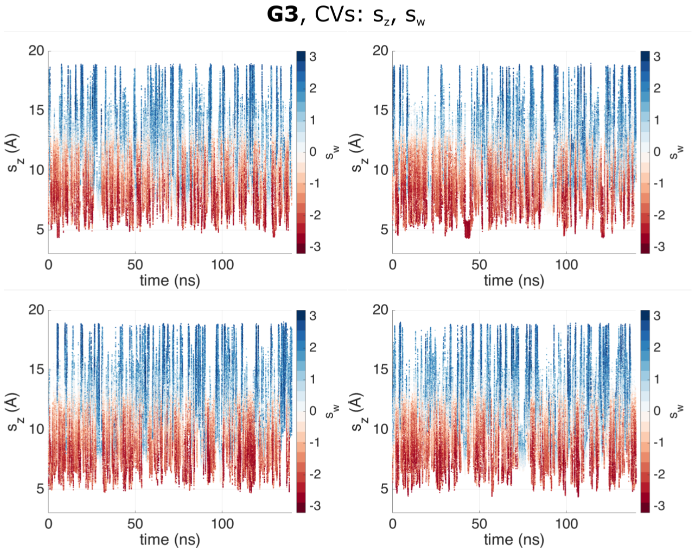

Supplementary Figure 43: **Deep-LDA OAH-G3 trajectories.** We show the dynamics of s_z in an OPES simulation where s_z and s_w are biased. The plot is coloured with the instantaneous value of s_w.

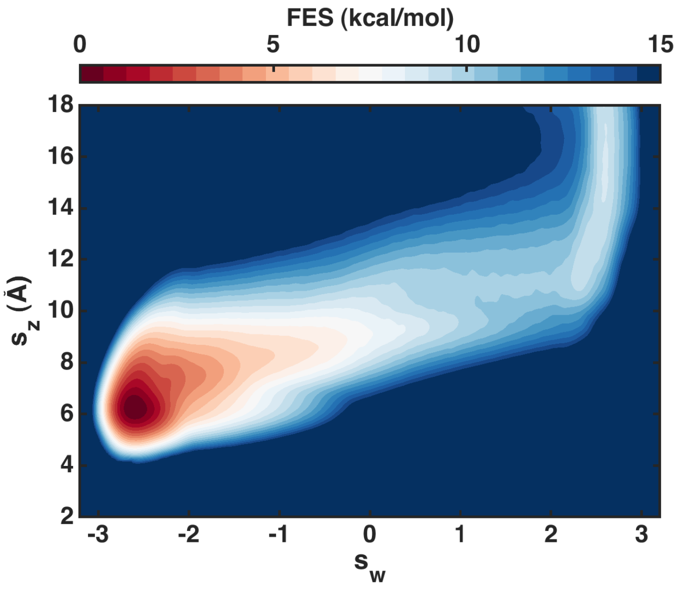

Supplementary Figure 44: **OAH-G3 FES with respect to** s_z **and** s_w.

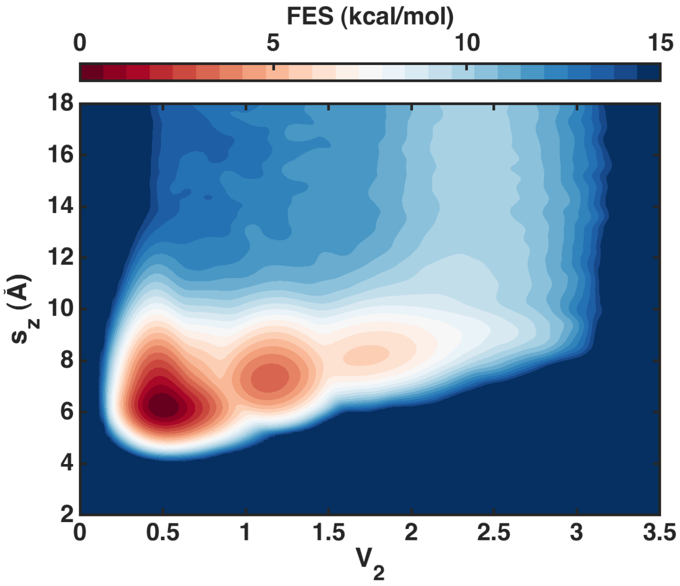

Supplementary Figure 45: **OAH-G3 FES with respect to s_z and V_2.**

OAH-G4

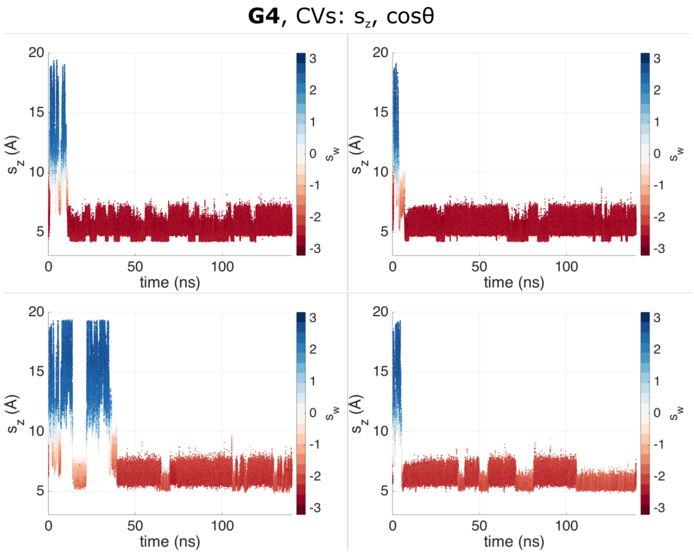

Supplementary Figure 46: **Standard OAH-G4 trajectories.** We show the dynamics of s_z in an OPES simulation where s_z and $\cos\theta$ are biased. The plot is coloured with the instantaneous value of s_w.

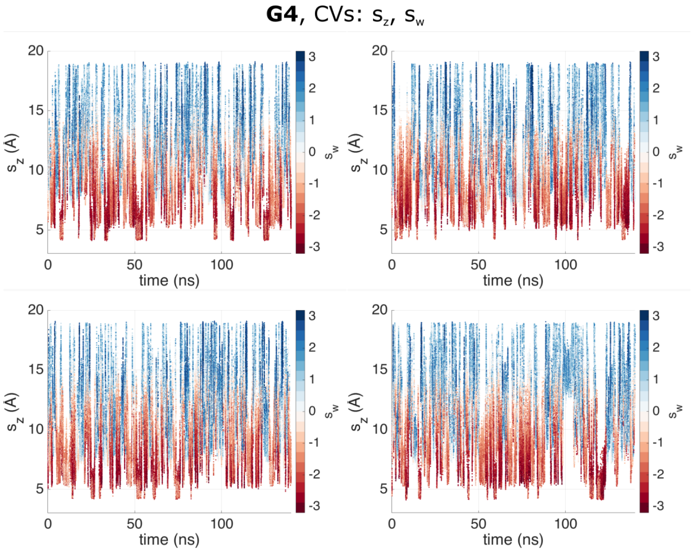

Supplementary Figure 47: **Deep-LDA OAH-G4 trajectories.** We show the dynamics of s_z in an OPES simulation where s_z and s_w are biased. The plot is coloured with the instantaneous value of s_w.

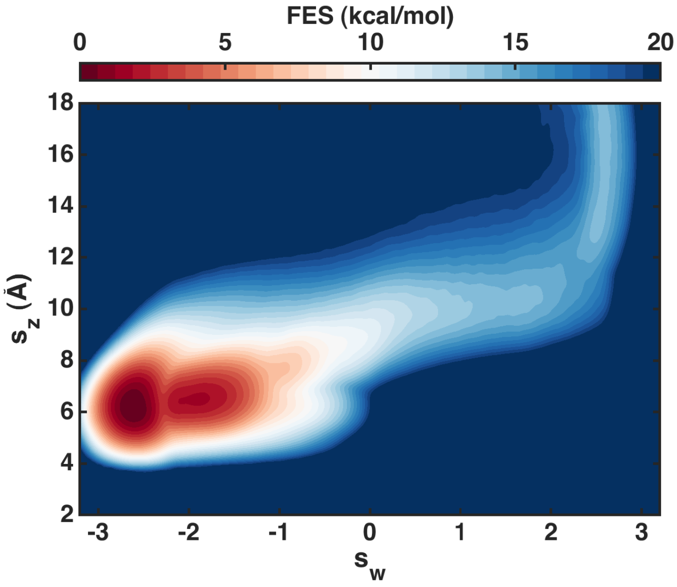

Supplementary Figure 48: **OAH-G4 FES with respect to** s_z **and** s_w.

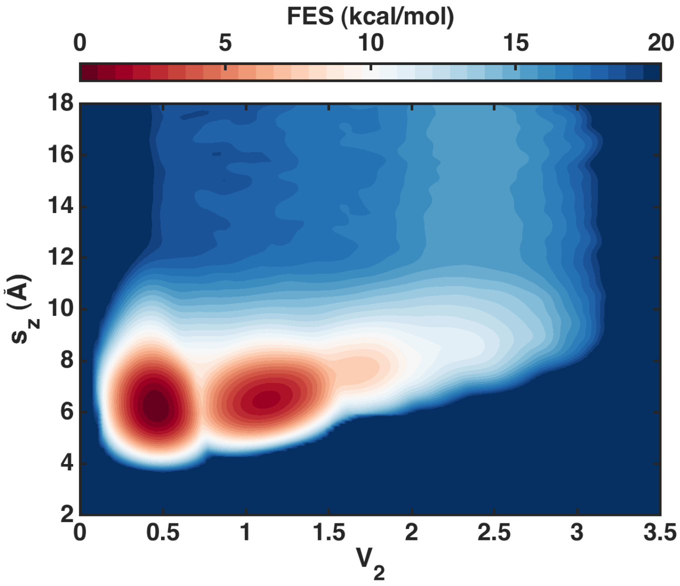

Supplementary Figure 49: **OAH-G4 FES with respect to s_z and V_2.**

OAH-G5

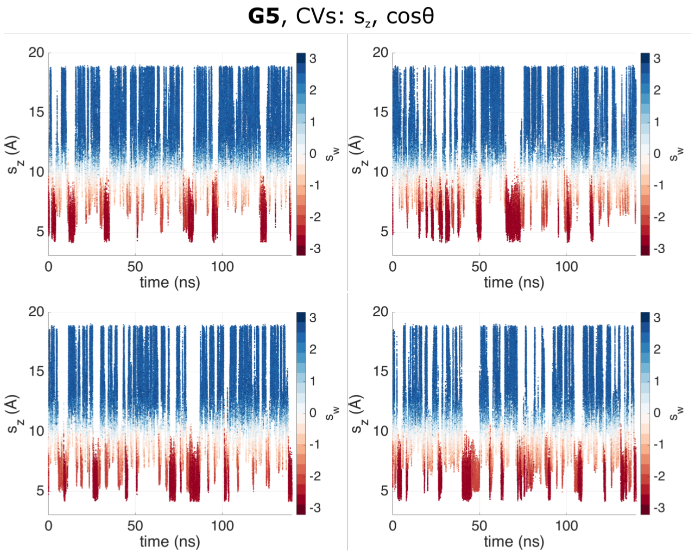

Supplementary Figure 50: **Standard OAH-G5 trajectories.** We show the dynamics of s_z in an OPES simulation where s_z and $\cos\theta$ are biased. The plot is coloured with the instantaneous value of s_{w}.

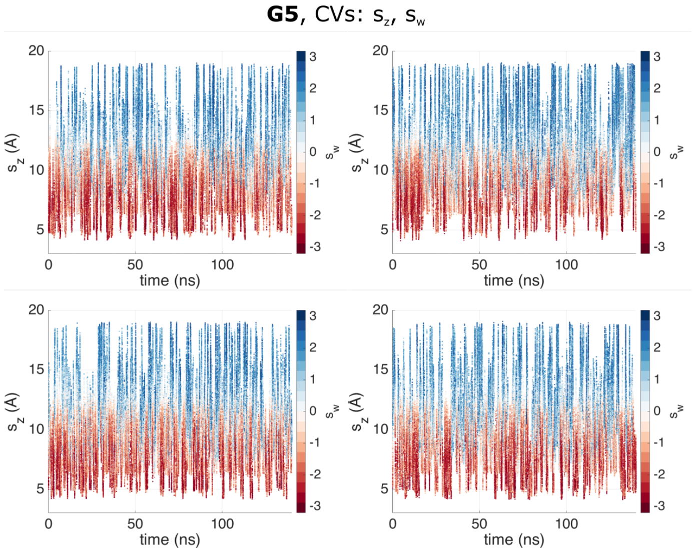

Supplementary Figure 51: **Deep-LDA OAH-G5 trajectories.** We show the dynamics of s_z in an OPES simulation where s_z and s_w are biased. The plot is coloured with the instantaneous value of s_w.

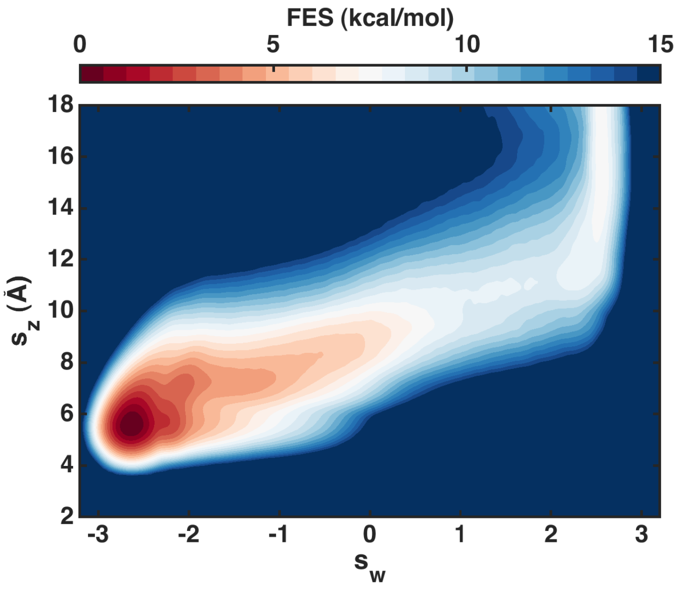

Supplementary Figure 52: **OAH-G5 FES with respect to** s_z **and** s_w.

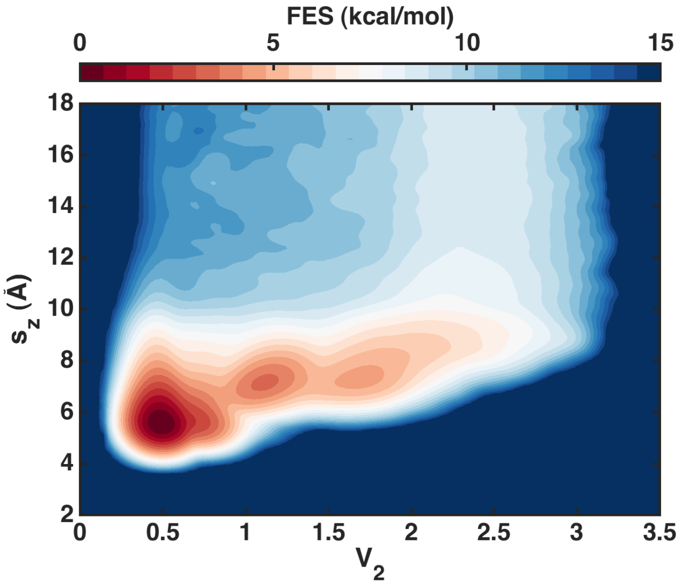

Supplementary Figure 53: **OAH-G5 FES with respect to s_z and V_2.**

OAH-G6

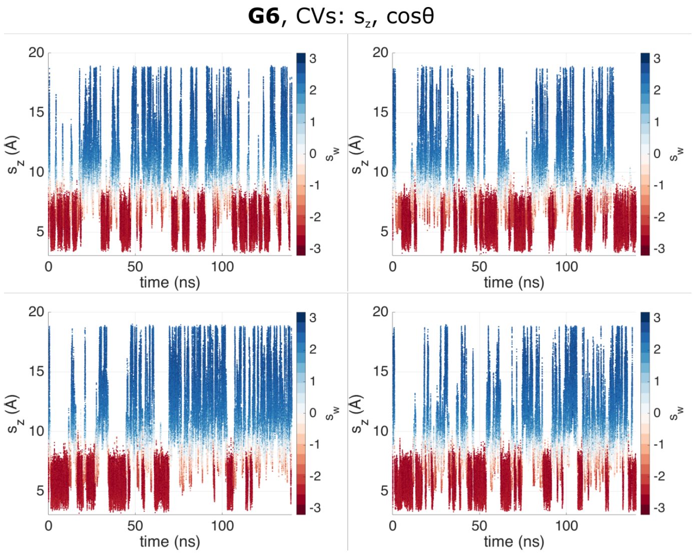

Supplementary Figure 54: **Standard OAH-G6 trajectories.** We show the dynamics of s_z in an OPES simulation where s_z and $\cos\theta$ are biased. The plot is coloured with the instantaneous value of s_w.

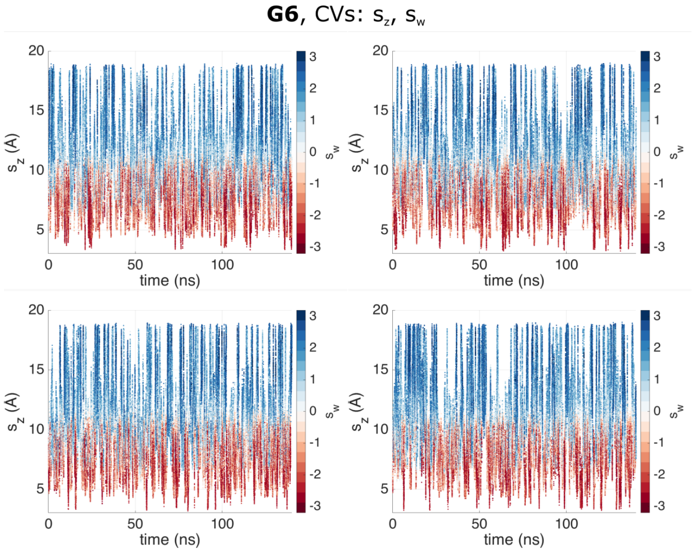

Supplementary Figure 55: **Deep-LDA OAH-G6 trajectories.** We show the dynamics of s_z in an OPES simulation where s_z and s_w are biased. The plot is coloured with the instantaneous value of s_w.

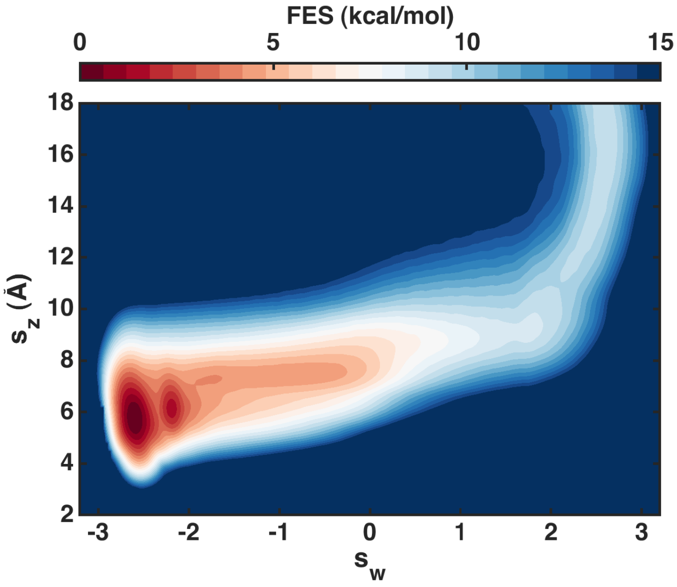

Supplementary Figure 56: **OAH-G6 FES with respect to s_z and s_w.**

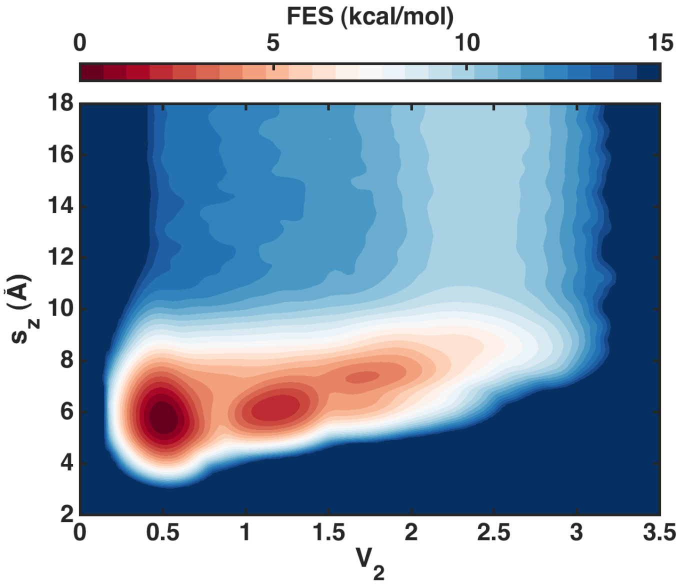

Supplementary Figure 57: **OAH-G6 FES with respect to s_z and** V_2**.**

All hosts

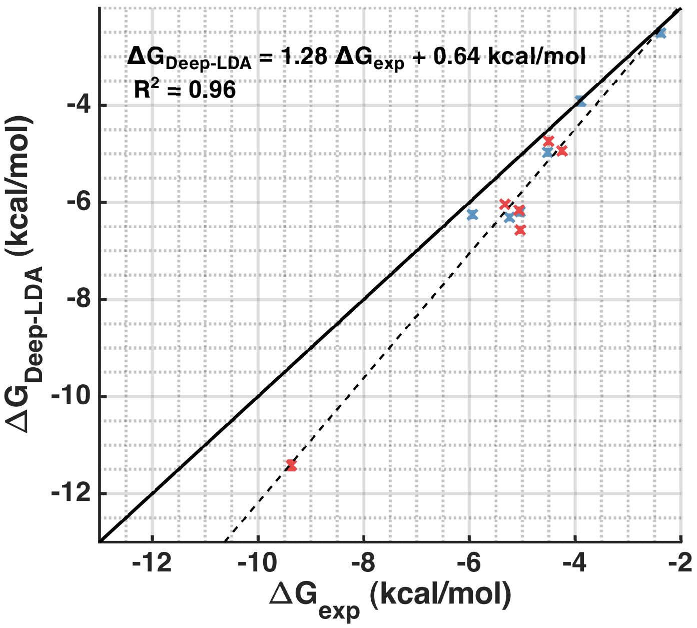

Supplementary Figure 58: **Comparison of the Deep-LDA binding free energies with experiments for both hosts.** We plot the value of ΔG obtained from the Deep-LDA simulations for every ligand and host versus the experimental values and show the corresponding linear fit. The values for host OAMe are shown in blue and the values for host OAH in red.

Host OAMe, TIP4P/EW water model

We have tested the Deep-LDA method against changing the water model from TIP3P to TIP4P/EW [18], performing simulations of the host system OAMe and all the six ligands. The results are presented in Supplementary Fig. 59-78 and Supplementary Tab. 19-26.

To setup the simulations, we first perform 10 ns of an NPT simulation, followed by 100 ps of NVT equilibration. Then, in complete analogy with what was described in the main text, we proceed with training three different Deep-LDA CV and run enhanced sampling OPES simulations.

While the binding binding free energies that we obtain are shifted by about 1.5 kcal mol^{-1} with respect to the ones from the TIP3P model simulations, the data is well aligned against experiments as can be seen in Supplementary Fig. 60. Modulo the shift, the quality of the linear fit is largely unchanged when compared to the TIP3P model results (see Supplementary Tab. 20). This shift in binding free energy can be attributed to factors such as a different solvation energy, different hydrogen bonds strengths and the effect of the water model on the host structure.

In analogy with the TIP3P calculations, for each guest molecule, we show the breakdown of the binding free energy for every NN CV, a set of trajectories and the 2-dimensional FES with respect to s_z, s_w and s_z, V_2 respectively.

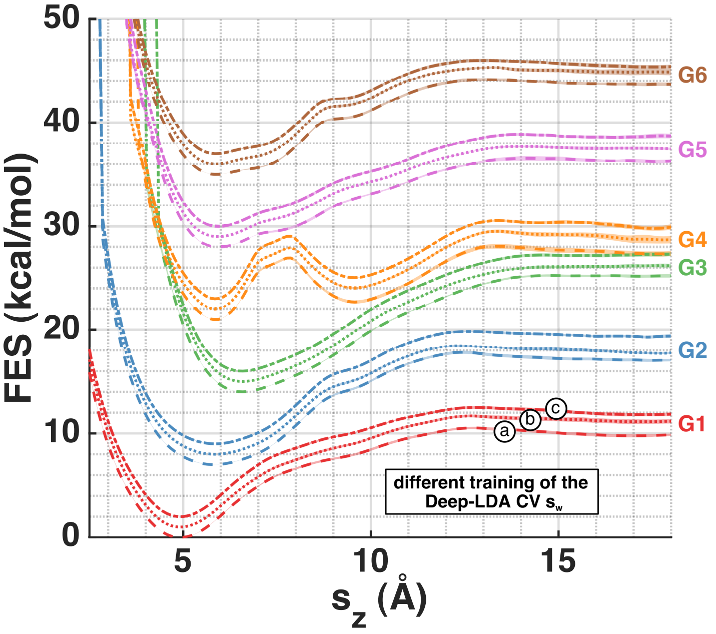

Supplementary Figure 59: **Free energy surfaces projected along the host-guest distance.** Same as Supplementary Fig. 3 for host OAMe and water model TIP4P/EW.

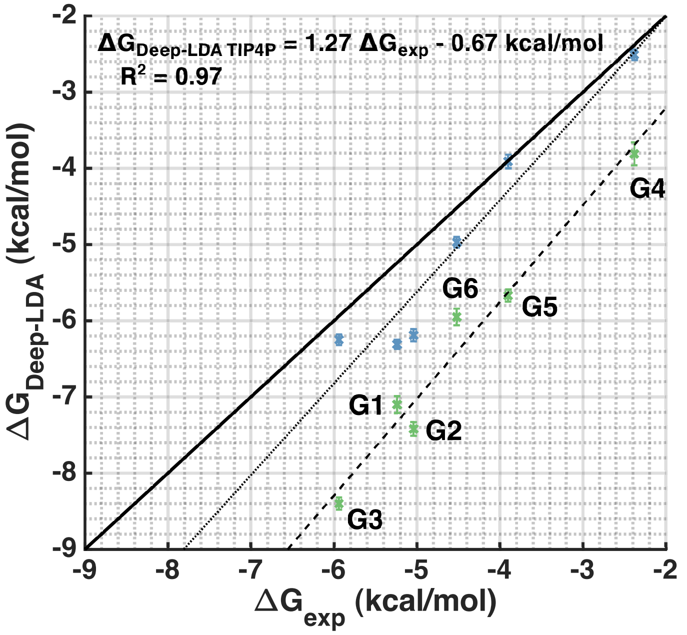

Supplementary Figure 60: **Comparison of the binding free energies with experiments and other calculations.** Same as Supplementary Fig. 4 for host OAMe and water model TIP4P/EW, with the data points shown in green and a dashed linear fit. For comparison's sake, the results corresponding to the TIP3P water model are shown with the data points in blue and a dotted linear fit.

OAMe-G1 TIP4P

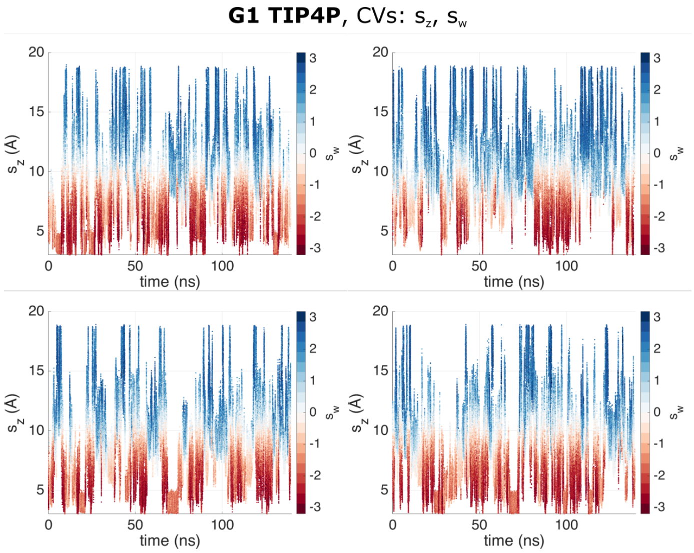

Supplementary Figure 61: **Deep-LDA OAMe-G1 trajectories with the TIP4P water model.** We show the dynamics of s_z in an OPES simulation where s_z and s_w are biased. The plot is coloured with the instantaneous value of s_w.

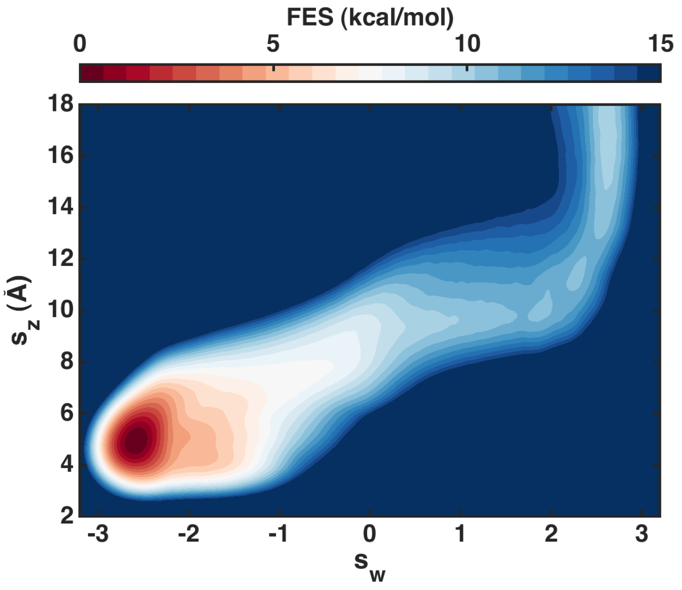

Supplementary Figure 62: **OAMe-G1 FES with respect to** s_z **and** s_w with water model TIP4P.

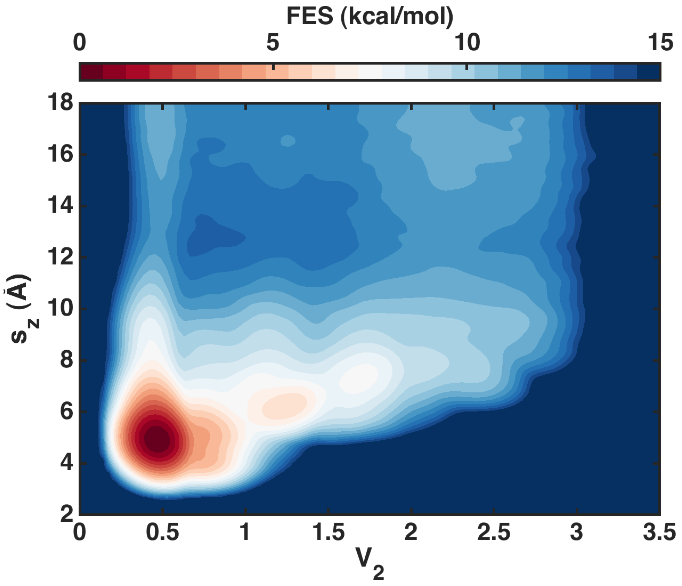

Supplementary Figure 63: **OAMe-G1 FES with respect to** s_z **and** V_2 **with water model TIP4P.**

OAMe-G2 TIP4P

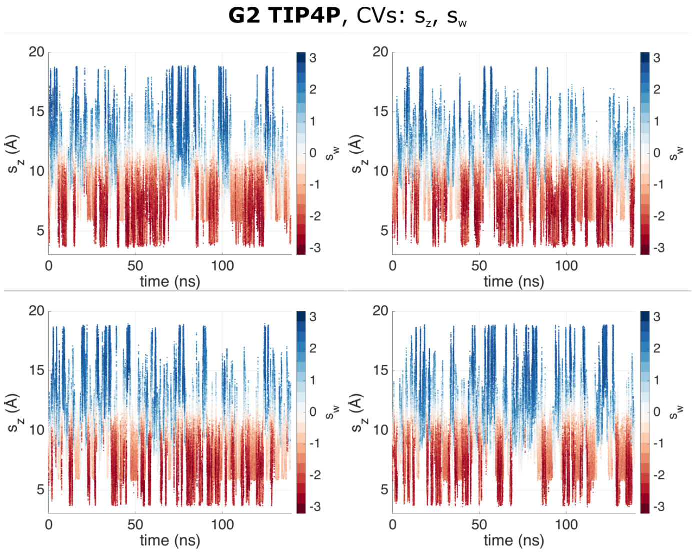

Supplementary Figure 64: **Deep-LDA OAMe-G2 trajectories with the TIP4P water model.** We show the dynamics of s_z in an OPES simulation where s_z and s_w are biased. The plot is coloured with the instantaneous value of s_w.

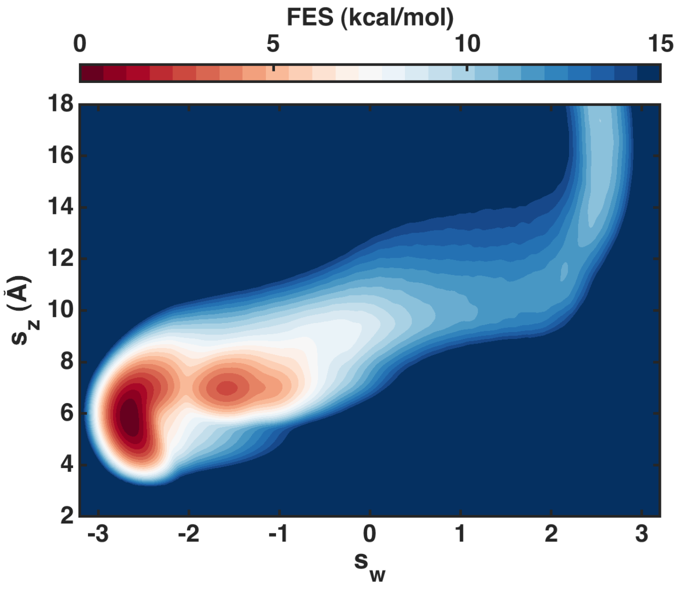

Supplementary Figure 65: **OAMe-G2 FES with respect to** s_z **and** s_w with water model TIP4P.

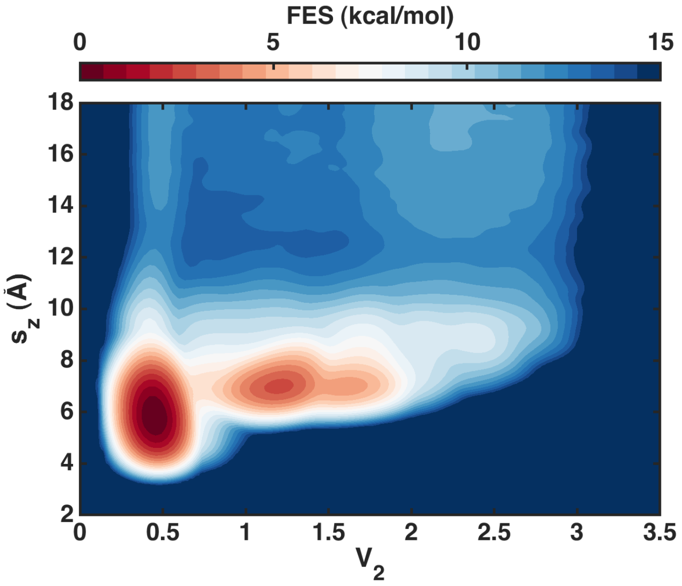

Supplementary Figure 66: **OAMe-G2 FES with respect to** s_z **and** V_2 **with water model TIP4P.**

OAMe-G3 TIP4P

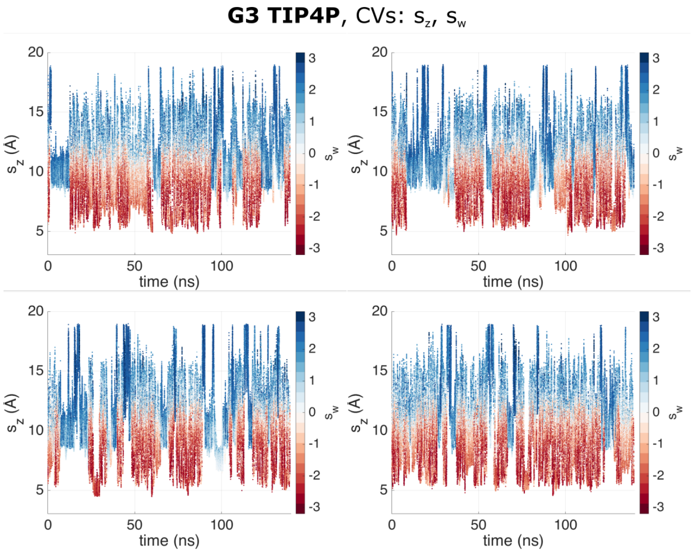

Supplementary Figure 67: **Deep-LDA OAMe-G3 trajectories with the TIP4P water model.** We show the dynamics of s_z in an OPES simulation where s_z and s_w are biased. The plot is coloured with the instantaneous value of s_w.

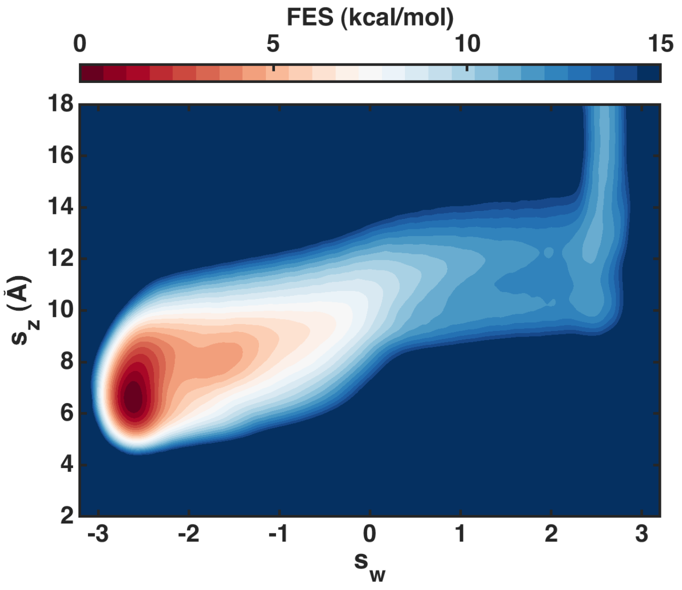

Supplementary Figure 68: **OAMe-G3 FES with respect to** s_z **and** s_w with water model TIP4P.

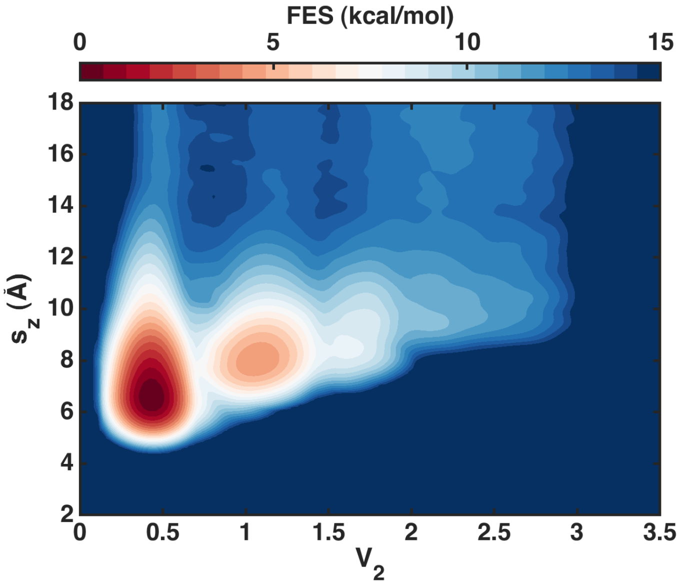

Supplementary Figure 69: **OAMe-G3 FES with respect to** s_z **and** V_2 **with water model TIP4P.**

OAMe-G4 TIP4P

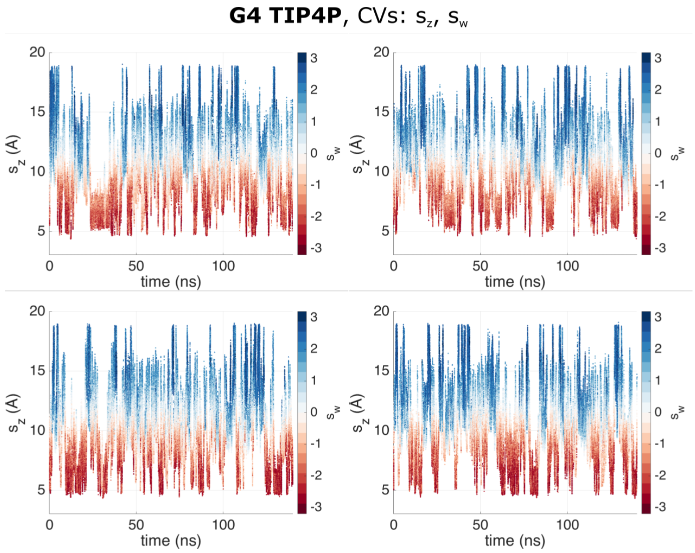

Supplementary Figure 70: **Deep-LDA OAMe-G4 trajectories with the TIP4P water model.** We show the dynamics of s_z in an OPES simulation where s_z and s_w are biased. The plot is coloured with the instantaneous value of s_W.

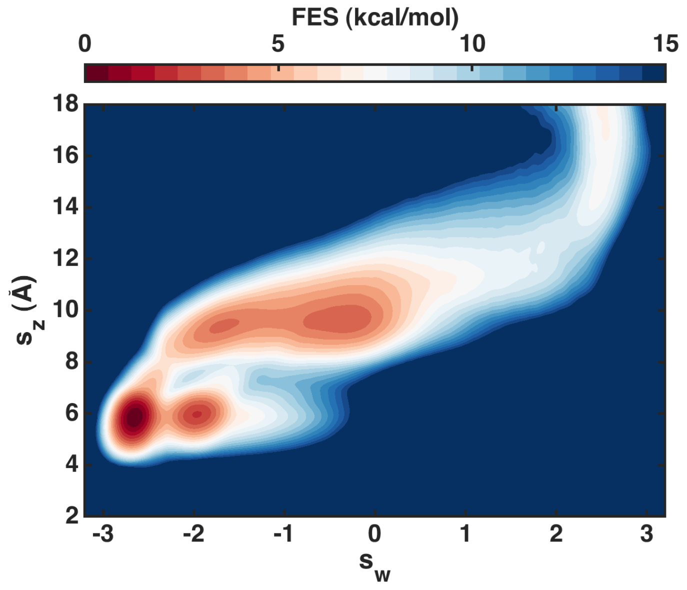

Supplementary Figure 71: **OAMe-G4 FES with respect to** s_z **and** s_w with water model TIP4P.

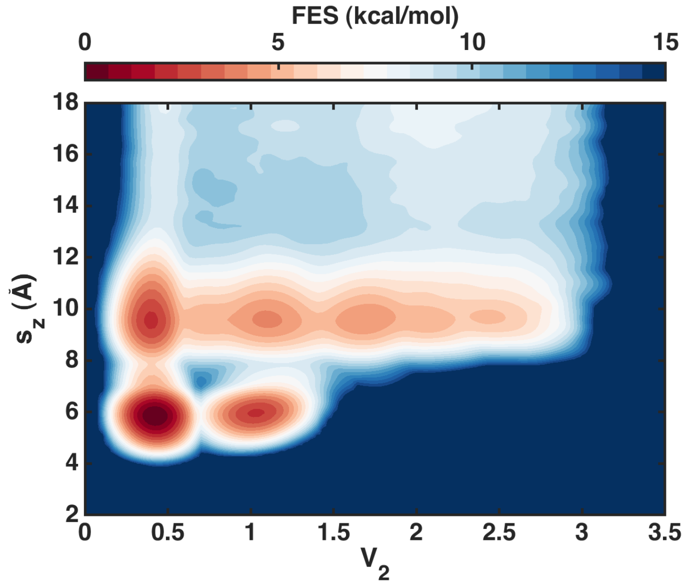

Supplementary Figure 72: **OAMe-G4 FES with respect to** s_z **and** V_2 **with water model TIP4P.**

OAMe-G5 TIP4P

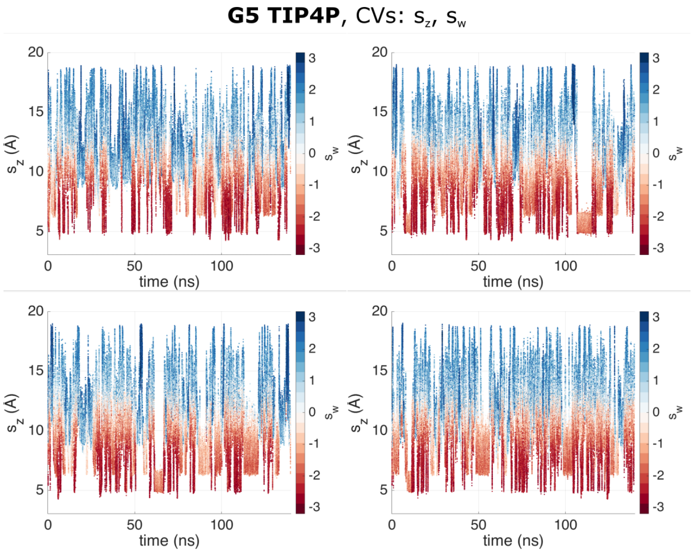

Supplementary Figure 73: **Deep-LDA OAMe-G5 trajectories with the TIP4P water model.** We show the dynamics of s_z in an OPES simulation where s_z and s_w are biased. The plot is coloured with the instantaneous value of s_w.

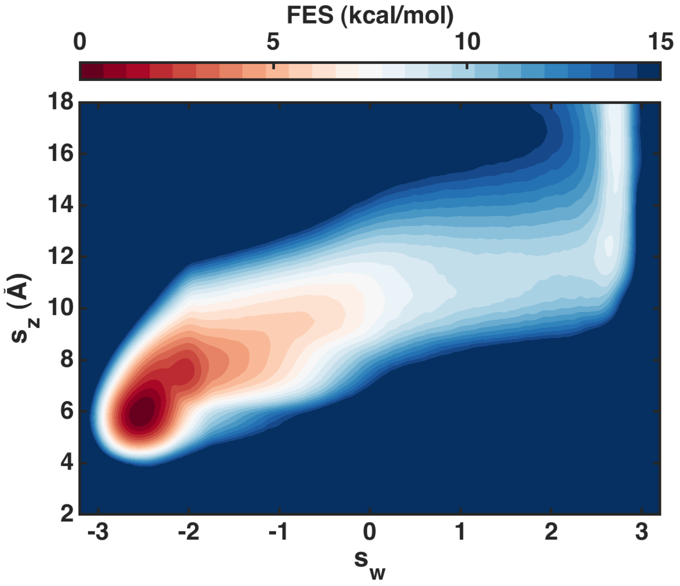

Supplementary Figure 74: **OAMe-G5 FES with respect to** s_z **and** s_w with water model TIP4P.

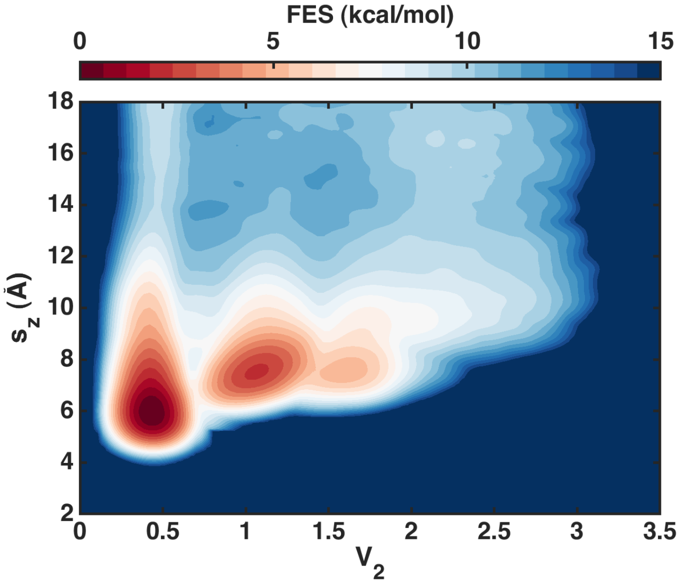

Supplementary Figure 75: **OAMe-G5 FES with respect to** s_z **and** V_2 **with water model TIP4P.**

OAMe-G6 TIP4P

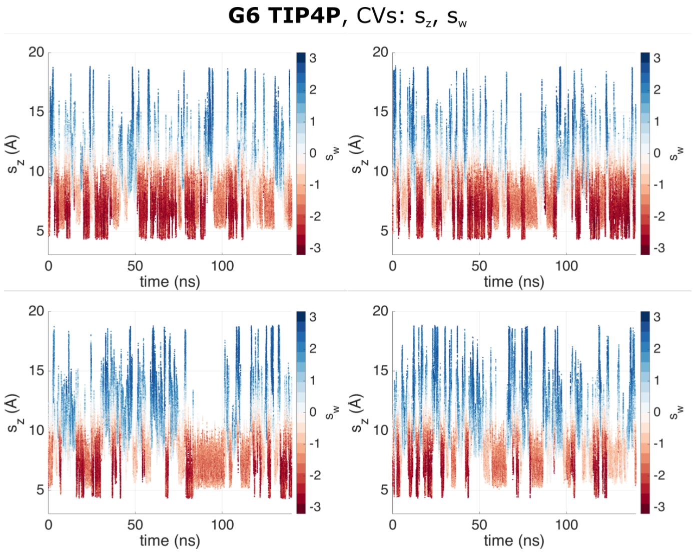

Supplementary Figure 76: **Deep-LDA OAMe-G6 trajectories with the TIP4P water model.** We show the dynamics of s_z in an OPES simulation where s_z and s_w are biased. The plot is coloured with the instantaneous value of s_w.

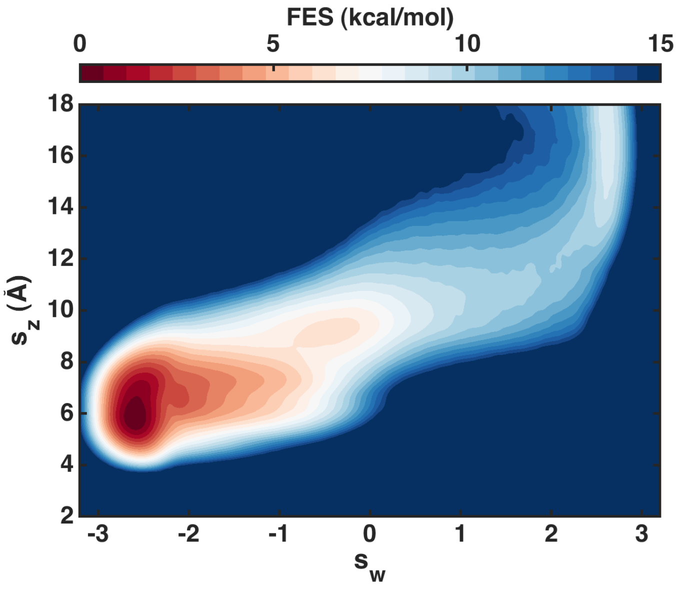

Supplementary Figure 77: **OAMe-G6 FES with respect to** s_z **and** s_w with water model TIP4P.

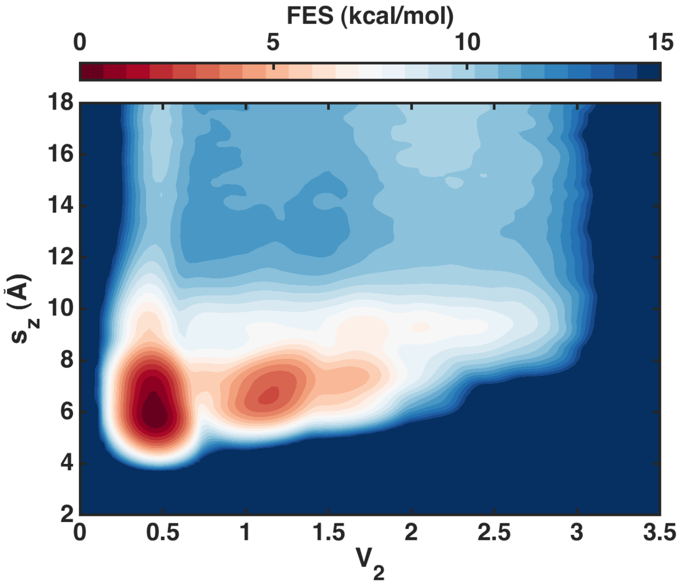

Supplementary Figure 78: **OAMe-G6 FES with respect to** s_z **and** V_2 **with water model TIP4P.**

Supplementary Tables

Host OAMe

Ligand Binding

Supplementary Table 1: **Binding free energies between the ligands and host OAMe.** We show the mean binding free energy ΔG (kcal mol^{-1}) for different simulation methods and compare them to the corresponding experimental value.

Ligand	Exp	Deep-LDA	Metadynamics	APR	SOMD-3
G1	-5.24	$\mathbf{-6.31 \pm 0.06}$	-6.36 ± 0.39	-7.61 ± 0.21	-6.78 ± 0.03
G2	-5.04	$\mathbf{-6.19 \pm 0.08}$	-6.76 ± 0.41	-6.99 ± 0.30	-7.59 ± 0.67
G3	-5.94	$\mathbf{-6.27 \pm 0.07}$	-5.44 ± 0.56	-7.34 ± 0.22	-7.42 ± 0.01
G4	-2.38	$\mathbf{-2.51 \pm 0.07}$	-3.64 ± 1.63	-1.99 ± 0.23	-3.36 ± 0.21
G5	-3.90	$\mathbf{-3.91 \pm 0.09}$	-3.45 ± 0.34	-5.30 ± 0.22	-6.69 ± 0.81
G6	-4.52	$\mathbf{-4.97 \pm 0.07}$	-4.28 ± 0.34	-6.02 ± 0.21	-5.61 ± 0.16

Supplementary Table 2: **Error statistics for host OAMe.** Following the procedure from [1], we provide the absolute and offset error of different simulation methods using the same model. RMSE is the root mean-squared error, R^2 is Pearson's coefficient of determination, m the linear regression slope and τ the Kendall rank correlation coefficient.

metric	method	RMSE	R^2	m	τ
absolute	Deep-LDA	**0.68** (**0.67 ± 0.18**)	**0.93** (**0.91 ± 0.13**)	**1.21** (**1.24 ± 0.29**)	**0.87** (**0.72 ± 0.21**)
absolute	Metadynamics	1.03 (1.21 ± 0.41)	0.53 (0.51 ± 0.29)	0.82 (0.97 ± 0.79)	0.47 (0.41 ± 0.31)
absolute	APR	1.62 (1.62 ± 0.24)	0.93 (0.89 ± 0.15)	1.62 (1.54 ± 0.40)	0.87 (0.72 ± 0.22)
absolute	SOMD-3	1.87 (1.88 ± 0.39)	0.78 (0.66 ± 0.30)	1.12 (1.01 ± 0.53)	0.60 (0.47 ± 0.30)
offset	Deep-LDA	**0.44** (**0.44 ± 0.07**)	**0.93** (**0.91 ± 0.13**)	**1.21** (**1.24 ± 0.29**)	**0.87** (**0.72 ± 0.22**)
offset	Metadynamics	0.90 (1.14 ± 0.33)	0.53 (0.51 ± 0.29)	0.82 (0.97 ± 0.84)	0.47 (0.40 ± 0.31)
offset	APR	0.86 (0.85 ± 0.28)	0.93 (0.89 ± 0.15)	1.62 (1.54 ± 0.40)	0.87 (0.72 ± 0.22)
offset	SOMD-3	0.69 (0.78 ± 0.26)	0.78 (0.66 ± 0.30)	1.12 (1.01 ± 0.52)	0.60 (0.47 ± 0.30)

OAMe-G1

Supplementary Table 3: **OAMe-G1 binding free energy breakdown.** Binding free energy ΔG (kcal mol^{-1}) of ligand G1 and its corresponding statistical weight w (a.u.) in every simulation block of calculations using Deep-LDA CVs from three different training.

Block	CV s_w^a		CV s_w^b		CV s_w^c	
	ΔG	w	ΔG	w	ΔG	w
1	-6.54	409	-6.48	331	-6.47	452
2	-6.30	592	-6.64	583	-6.40	529
3	-5.76	421	-6.45	598	-6.16	407
4	-6.19	459	-6.01	263	-6.33	449
5	-6.00	360	-6.19	370	-6.41	434
all	$\mathbf{-6.17} \pm 0.13$		$\mathbf{-6.41} \pm 0.11$		$\mathbf{-6.36} \pm 0.05$	

OAMe-G2

Supplementary Table 4: **OAMe-G2 binding free energy breakdown.** Binding free energy ΔG (kcal mol^{-1}) of ligand G1 and its corresponding statistical weight w (a.u.) in every simulation block of calculations using Deep-LDA CVs from three different training.

Block	CV s^a_w		CV s^b_w		CV s^c_w	
	ΔG	w	ΔG	w	ΔG	w
1	-6.50	585	-5.88	376	-6.58	781
2	-5.89	373	-6.08	446	-5.94	463
3	-6.25	493	-6.06	415	-5.74	307
4	-6.02	406	-6.46	425	-6.50	591
5	-6.01	391	-6.49	491	-5.56	356
all	$-\mathbf{6.17} \pm 0.12$		$-\mathbf{6.21} \pm 0.12$		$-\mathbf{6.19} \pm 0.22$	

OAMe-G3

Supplementary Table 5: **OAMe-G3 binding free energy breakdown.** Binding free energy ΔG (kcal mol^{-1}) of ligand G1 and its corresponding statistical weight w (a.u.) in every simulation block of calculations using Deep-LDA CVs from three different training.

Block	CV s^a_w		CV s^b_w		CV s^c_w	
	ΔG	w	ΔG	w	ΔG	w
1	-6.31	526	-6.58	368	-5.43	305
2	-6.14	380	-6.07	283	-6.01	381
3	-6.24	569	-6.30	440	-6.48	456
4	-6.53	407	-6.41	350	-6.19	447
5	-6.46	596	-5.97	242	-6.39	459
all	$-\mathbf{6.34} \pm 0.07$		$-\mathbf{6.30} \pm 0.11$		$-\mathbf{6.15} \pm 0.17$	

OAMe-G4

Supplementary Table 6: **OAMe-G4 binding free energy breakdown.** Binding free energy ΔG (kcal mol^{-1}) of ligand G1 and its corresponding statistical weight w (a.u.) in every simulation block of calculations using Deep-LDA CVs from three different training.

Block	CV s^a_w		CV s^b_w		CV s^c_w	
	ΔG	w	ΔG	w	ΔG	w
1	-2.53	356	-2.94	476	-2.41	468
2	-2.40	377	-1.95	231	-2.12	278
3	-2.39	296	-2.58	481	-2.38	299
4	-2.80	305	-2.55	349	-2.17	205
5	-2.45	251	-2.63	396	-3.07	197
all	$-\mathbf{2.51} \pm 0.08$		$-\mathbf{2.60} \pm 0.15$		$-\mathbf{2.40} \pm 0.15$	

OAMe-G5

Supplementary Table 7: **OAMe-G5 binding free energy breakdown.** Binding free energy ΔG (kcal mol^{-1}) of ligand G1 and its corresponding statistical weight w (a.u.) in every simulation block of calculations using Deep-LDA CVs from three different training.

Block	CV s_w^a		CV s_w^b		CV s_w^c	
	ΔG	w	ΔG	w	ΔG	w
1	-3.69	269	-3.54	334	-3.71	326
2	-3.97	396	-3.83	412	-3.96	441
3	-4.10	378	-3.06	265	-4.18	479
4	-4.11	398	-3.43	326	-4.15	525
5	-4.45	374	-4.13	395	-3.68	426
all	$\mathbf{-4.09} \pm 0.12$		$\mathbf{-3.65} \pm 0.18$		$\mathbf{-3.96} \pm 0.11$	

OAMe-G6

Supplementary Table 8: **OAMe-G6 binding free energy breakdown.** Binding free energy ΔG (kcal mol^{-1}) of ligand G1 and its corresponding statistical weight w (a.u.) in every simulation block of calculations using Deep-LDA CVs from three different training.

Block	CV s_w^a		CV s_w^b		CV s_w^c	
	ΔG	w	ΔG	w	ΔG	w
1	-4.84	438	-5.05	469	-4.78	213
2	-5.08	525	-5.37	701	-5.06	487
3	-4.92	405	-4.93	412	-5.01	420
4	-4.79	443	-4.53	395	-5.32	586
5	-5.01	531	-4.25	256	-4.70	370
all blocks	$\mathbf{-4.94} \pm 0.05$		$\mathbf{-4.94} \pm 0.20$		$\mathbf{-5.03} \pm 0.12$	

Host OAH

Ligand Binding

Supplementary Table 9: **Binding free energies between the ligands and host OAH.** We show the mean binding free energy ΔG (kcal mol^{-1}) for different simulation methods and compare them to the corresponding experimental value.

Ligand	Exp	Deep-LDA	Metadynamics	APR	SOMD-3
G1	-5.04	$\mathbf{-6.57 \pm 0.06}$	-6.07 ± 0.35	-6.50 ± 0.23	-6.94 ± 0.30
G2	-4.25	$\mathbf{-4.94 \pm 0.06}$	-5.08 ± 0.72	-5.42 ± 0.22	-5.23 ± 0.15
G3	-5.06	$\mathbf{-6.16 \pm 0.05}$	-5.92 ± 0.58	-6.82 ± 0.23	-7.28 ± 0.14
G4	-9.37	$\mathbf{-11.42 \pm 0.10}$	-11.85 ± 0.61	-12.34 ± 0.23	-12.79 ± 1.18
G5	-4.50	$\mathbf{-4.74 \pm 0.06}$	-4.80 ± 0.46	-4.77 ± 0.24	-7.70 ± 0.37
G6	-5.33	$\mathbf{-6.03 \pm 0.04}$	-5.52 ± 0.64	-6.46 ± 0.23	-6.16 ± 0.10

Supplementary Table 10: **Error statistics for host OAH.** Following the procedure from [1], we provide the absolute and offset error of different simulation methods using the same model.

metric	method	RMSE	R^2	m	τ
absolute	Deep-LDA	**1.21** (**1.19** ± **0.25**)	**0.97** (**0.91** ± **0.13**)	**1.28** (**1.33** ± **0.83**)	**0.47** (**0.40** ± **0.30**)
absolute	Metadynamics	1.21 (1.28 ± 0.40)	0.98 (0.76 ± 0.32)	1.38 (1.21 ± 2.48)	0.47 (0.44 ± 0.32)
absolute	APR	1.67 (1.65 ± 0.36)	0.97 (0.88 ± 0.18)	1.41 (1.49 ± 0.80)	0.60 (0.55 ± 0.25)
absolute	SOMD-3	2.31 (2.32 ± 0.50)	0.88 (0.73 ± 0.31)	1.32 (1.00 ± 1.39)	0.33 (0.28 ± 0.39)
offset	Deep-LDA	**0.60** (**0.59** ± **0.13**)	**0.97** (**0.91** ± **0.13**)	**1.28** (**1.33** ± **0.79**)	**0.47** (**0.40** ± **0.30**)
offset	Metadynamics	0.75 (0.90 ± 0.29)	0.98 (0.76 ± 0.32)	1.38 (1.20 ± 1.24)	0.47 (0.44 ± 0.32)
offset	APR	0.81 (0.81 ± 0.25)	0.97 (0.88 ± 0.18)	1.41 (1.49 ± 0.95)	0.60 (0.55 ± 0.25)
offset	SOMD-3	0.99 (1.08 ± 0.31)	0.88 (0.73 ± 0.31)	1.3 (1.0 ± 2.0)	0.33 (0.28 ± 0.39)

OAH-G1

Supplementary Table 11: **OAH-G1 binding free energy breakdown.** Binding free energy ΔG (kcal mol^{-1}) of ligand G1 and its corresponding statistical weight w (a.u.) in every simulation block of calculations using Deep-LDA CVs from three different training.

Block	CV s_w^a		CV s_w^b		CV s_w^c	
	ΔG	w	ΔG	w	ΔG	w
1	-6.42	35	-6.15	33	-6.89	47
2	-6.15	30	-6.50	39	-6.94	50
3	-6.70	56	-6.45	38	-6.35	39
4	-6.62	42	-6.60	47	-6.90	43
5	-6.52	36	-6.61	36	-6.49	38
all	$\mathbf{-6.52} \pm 0.09$		$\mathbf{-6.48} \pm 0.08$		$\mathbf{-6.74} \pm 0.12$	

OAH-G2

Supplementary Table 12: **OAH-G2 binding free energy breakdown.** Binding free energy ΔG (kcal mol^{-1}) of ligand G1 and its corresponding statistical weight w (a.u.) in every simulation block of calculations using Deep-LDA CVs from three different training.

Block	CV s_w^a		CV s_w^b		CV s_w^c	
	ΔG	w	ΔG	w	ΔG	w
1	-4.67	35	-4.83	40	-4.83	27
2	-5.12	45	-4.70	33	-5.41	47
3	-4.97	35	-5.00	42	-4.87	37
4	-4.75	24	-4.87	36	-5.01	42
5	-5.06	36	-4.70	33	-5.13	34
all	$\mathbf{-4.94} \pm 0.09$		$\mathbf{-4.83} \pm 0.06$		$\mathbf{-5.08} \pm 0.11$	

OAH-G3

Supplementary Table 13: **OAH-G3 binding free energy breakdown.** Binding free energy ΔG (kcal mol^{-1}) of ligand G1 and its corresponding statistical weight w (a.u.) in every simulation block of calculations using Deep-LDA CVs from three different training.

Block	CV s_w^a		CV s_w^b		CV s_w^c	
	ΔG	w	ΔG	w	ΔG	w
1	-5.90	43	-5.91	31	-6.12	45
2	-6.12	46	-6.93	53	-6.19	48
3	-5.91	45	-6.24	41	-6.06	42
4	-6.08	44	-5.88	32	-6.26	39
5	-5.98	37	-6.22	43	-6.24	41
all	$-\mathbf{6.00} \pm 0.05$		$-\mathbf{6.31} \pm 0.20$		$-\mathbf{6.17} \pm 0.04$	

OAH-G4

Supplementary Table 14: **OAH-G4 binding free energy breakdown.** Binding free energy ΔG (kcal mol^{-1}) of ligand G1 and its corresponding statistical weight w (a.u.) in every simulation block of calculations using Deep-LDA CVs from three different training.

Block	CV s_w^a		CV s_w^b		CV s_w^c	
	ΔG	w	ΔG	w	ΔG	w
1	-11.53	48	-11.11	33	-11.21	33
2	-11.38	35	-10.78	24	-11.80	44
3	-11.56	32	-11.03	25	-11.45	42
4	-12.28	60	-11.21	31	-11.24	26
5	-11.45	36	-11.22	44	-11.44	21
all	$-\mathbf{11.71} \pm 0.19$		$-\mathbf{11.10} \pm 0.08$		$-\mathbf{11.46} \pm 0.12$	

OAH-G5

Supplementary Table 15: **OAH-G5 binding free energy breakdown.** Binding free energy ΔG (kcal mol^{-1}) of ligand G1 and its corresponding statistical weight w (a.u.) in every simulation block of calculations using Deep-LDA CVs from three different training.

Block	CV s_w^a		CV s_w^b		CV s_w^c	
	ΔG	w	ΔG	w	ΔG	w
1	-5.09	43	-4.80	41	-4.70	53
2	-4.74	46	-4.35	29	-4.42	32
3	-5.01	45	-4.86	55	-4.71	46
4	-4.56	44	-4.56	38	-4.98	51
5	-4.80	37	-4.59	40	-4.51	49
all	$-\mathbf{4.85} \pm 0.10$		$-\mathbf{4.67} \pm 0.09$		$-\mathbf{4.68} \pm 0.10$	

OAH-G6

Supplementary Table 16: **OAH-G6 binding free energy breakdown.** Binding free energy ΔG (kcal mol^{-1}) of ligand G1 and its corresponding statistical weight w (a.u.) in every simulation block of calculations using Deep-LDA CVs from three different training.

Block	CV s_w^a		CV s_w^b		CV s_w^c	
	ΔG	w	ΔG	w	ΔG	w
1	-6.20	43	-5.96	55	-6.26	45
2	-6.17	46	-5.77	32	-6.22	50
3	-6.13	45	-5.78	28	-6.09	47
4	-5.93	44	-6.10	52	-5.99	40
5	-5.98	37	-5.79	38	-6.02	37
all	$\mathbf{-6.10} \pm 0.05$		$\mathbf{-5.87} \pm 0.04$		$\mathbf{-6.10} \pm 0.04$	

All hosts

Supplementary Table 17: **Binding free energies summary between the ligands and hosts OAMe and OAH.** We show the mean binding free energy ΔG (kcal mol^{-1}) for different simulation methods and compare them to the corresponding experimental value.

Host	Ligand	Exp	Deep-LDA	Metadynamics	APR	SOMD-3
OAMe	G1	-5.24	$\mathbf{-6.31 \pm 0.06}$	-6.36 ± 0.39	-7.61 ± 0.21	-6.78 ± 0.03
OAMe	G2	-5.04	$\mathbf{-6.19 \pm 0.08}$	-6.76 ± 0.41	-6.99 ± 0.30	-7.59 ± 0.67
OAMe	G3	-5.94	$\mathbf{-6.27 \pm 0.07}$	-5.44 ± 0.56	-7.34 ± 0.22	-7.42 ± 0.01
OAMe	G4	-2.38	$\mathbf{-2.51 \pm 0.07}$	-3.64 ± 1.63	-1.99 ± 0.23	-3.36 ± 0.21
OAMe	G5	-3.90	$\mathbf{-3.91 \pm 0.09}$	-3.45 ± 0.34	-5.30 ± 0.22	-6.69 ± 0.81
OAMe	G6	-4.52	$\mathbf{-4.97 \pm 0.07}$	-4.28 ± 0.34	-6.02 ± 0.21	-5.61 ± 0.16
OAH	G1	-5.04	$\mathbf{-6.57 \pm 0.06}$	-6.07 ± 0.35	-6.50 ± 0.23	-6.94 ± 0.30
OAH	G2	-4.25	$\mathbf{-4.94 \pm 0.06}$	-5.08 ± 0.72	-5.42 ± 0.22	-5.23 ± 0.15
OAH	G3	-5.06	$\mathbf{-6.16 \pm 0.05}$	-5.92 ± 0.58	-6.82 ± 0.23	-7.28 ± 0.14
OAH	G4	-9.37	$\mathbf{-11.42 \pm 0.10}$	-11.85 ± 0.61	-12.34 ± 0.23	-12.79 ± 1.18
OAH	G5	-4.50	$\mathbf{-4.74 \pm 0.06}$	-4.80 ± 0.46	-4.77 ± 0.24	-7.70 ± 0.37
OAH	G6	-5.33	$\mathbf{-6.03 \pm 0.04}$	-5.52 ± 0.64	-6.46 ± 0.23	-6.16 ± 0.10

Supplementary Table 18: **Error statistics summary for hosts OAMe and OAH.** Following the procedure from [1], we provide the absolute and offset error of different simulation methods using the same model.

metric	method	RMSE	R^2	m	τ
absolute	Deep-LDA	$\mathbf{0.98\ (0.97 \pm 0.18)}$	$\mathbf{0.96\ (0.93 \pm 0.08)}$	$\mathbf{1.28\ (1.29 \pm 0.13)}$	$\mathbf{0.70\ (0.62 \pm 0.17)}$
absolute	Metadynamics	$1.12\ (1.28 \pm 0.29)$	$0.86\ (0.70 \pm 0.26)$	$1.24\ (1.17 \pm 0.39)$	$0.48\ (0.47 \pm 0.19)$
absolute	APR	$1.65\ (1.65 \pm 0.21)$	$0.95\ (0.90 \pm 0.10)$	$1.43\ (1.45 \pm 0.17)$	$0.73\ (0.68 \pm 0.13)$
absolute	SOMD-3	$2.1\ (2.13 \pm 0.33)$	$0.87\ (0.74 \pm 0.24)$	$1.26\ (1.17 \pm 0.33)$	$0.39\ (0.39 \pm 0.23)$
offset	Deep-LDA	$\mathbf{0.52\ (0.52 \pm 0.08)}$	$\mathbf{0.96\ (0.93 \pm 0.08)}$	$\mathbf{1.22\ (1.24 \pm 0.12)}$	$\mathbf{0.73\ (0.68 \pm 0.15)}$
offset	Metadynamics	$0.83\ (1.05 \pm 0.23)$	$0.85\ (0.68 \pm 0.26)$	$1.19\ (1.13 \pm 0.38)$	$0.52\ (0.50 \pm 0.19)$
offset	APR	$0.84\ (0.85 \pm 0.18)$	$0.94\ (0.90 \pm 0.11)$	$1.42\ (1.44 \pm 0.17)$	$0.73\ (0.68 \pm 0.13)$
offset	SOMD-3	$0.85\ (0.96 \pm 0.22)$	$0.86\ (0.73 \pm 0.24)$	$1.23\ (1.13 \pm 0.31)$	$0.45\ (0.41 \pm 0.23)$

Host OAMe, TIP4P/EW water model

Supplementary Table 19: **Binding free energies between the ligands and host OAMe with water model TIP4P.** We show the mean binding free energy ΔG (kcal mol^{-1}) for Deep-LDA simulations with water model TIP4P/EW and compare them to the corresponding experimental value and the Deep-LDA results with water model TIP3P.

Ligand	Exp	Deep-LDA TIP3P	Deep-LDA TIP4P
G1	-5.24	-6.31 ± 0.06	$\mathbf{-7.10 \pm 0.11}$
G2	-5.04	-6.19 ± 0.08	$\mathbf{-7.42 \pm 0.09}$
G3	-5.94	-6.27 ± 0.07	$\mathbf{-8.40 \pm 0.08}$
G4	-2.38	-2.51 ± 0.07	$\mathbf{-3.81 \pm 0.15}$
G5	-3.90	-3.91 ± 0.09	$\mathbf{-5.67 \pm 0.08}$
G6	-4.52	-4.97 ± 0.07	$\mathbf{-5.95 \pm 0.11}$

Supplementary Table 20: **Error statistics for host OAMe and water model TIP4P.** Following the procedure from [1], we provide the absolute and offset error of Deep-LDA simulations with water model TIP4P/EW compared to simulations using water model TIP3P.

metric	method	RMSE	R^2	m	τ
absolute	Deep-LDA TIP4P	$\mathbf{1.93}$ $(\mathbf{1.93 \pm 0.18})$	$\mathbf{0.97}$ $(\mathbf{0.95 \pm 0.06})$	$\mathbf{1.27}$ $(\mathbf{1.30 \pm 0.20})$	$\mathbf{0.87}$ $(\mathbf{0.72 \pm 0.21})$
absolute	Deep-LDA TIP3P	0.68 (0.67 ± 0.18)	0.93 (0.91 ± 0.13)	1.21 (1.24 ± 0.29)	0.87 (0.72 ± 0.21)
offset	Deep-LDA TIP4P	$\mathbf{0.41}$ $(\mathbf{0.42 \pm 0.08})$	$\mathbf{0.97}$ $(\mathbf{0.95 \pm 0.06})$	$\mathbf{1.27}$ $(\mathbf{1.30 \pm 0.20})$	$\mathbf{0.87}$ $(\mathbf{0.72 \pm 0.22})$
offset	Deep-LDA TIP3P	0.44 (0.44 ± 0.07)	0.93 (0.91 ± 0.13)	1.21 (1.24 ± 0.29)	0.87 (0.72 ± 0.22)

OAMe-G1 TIP4P

Supplementary Table 21: **OAMe-G1 with water model TIP4P binding free energy breakdown.** Binding free energy ΔG (kcal mol^{-1}) of ligand G1 and its corresponding statistical weight w (a.u.) in every simulation block of calculations using Deep-LDA CVs from three different training.

Block	CV s_w^a		CV s_w^b		CV s_w^c	
	ΔG	w	ΔG	w	ΔG	w
1	-6.65	25	-6.53	22	-6.82	35
2	-7.01	49	-7.06	28	-6.55	31
3	-7.29	52	-7.33	29	-6.83	28
4	-6.53	32	-7.71	61	-7.54	47
5	-7.03	33	-7.63	31	-6.97	34
all	$\mathbf{-6.96} \pm 0.14$		$\mathbf{-7.37} \pm 0.22$		$\mathbf{-7.00} \pm 0.18$	

OAMe-G2 TIP4P

Supplementary Table 22: **OAMe-G2 with water model TIP4P binding free energy breakdown.** Binding free energy ΔG (kcal mol^{-1}) of ligand G1 and its corresponding statistical weight w (a.u.) in every simulation block of calculations using Deep-LDA CVs from three different training.

Block	CV s_w^a		CV s_w^b		CV s_w^c	
	ΔG	w	ΔG	w	ΔG	w
1	-7.54	44	-7.15	44	-7.81	46
2	-7.74	51	-7.31	61	-7.73	40
3	-6.97	37	-7.11	48	-7.81	40
4	-7.48	55	-7.51	51	-7.82	44
5	-7.18	35	-6.80	46	-7.21	41
all	$\mathbf{-7.42} \pm 0.13$		$\mathbf{-7.19} \pm 0.12$		$\mathbf{-7.68} \pm 0.12$	

OAMe-G3 TIP4P

Supplementary Table 23: **OAMe-G3 with water model TIP4P binding free energy breakdown.** Binding free energy ΔG (kcal mol^{-1}) of ligand G1 and its corresponding statistical weight w (a.u.) in every simulation block of calculations using Deep-LDA CVs from three different training.

Block	CV s_w^a		CV s_w^b		CV s_w^c	
	ΔG	w	ΔG	w	ΔG	w
1	-8.24	42	-8.48	40	-8.47	43
2	-8.40	41	-8.76	53	-7.82	27
3	-8.83	59	-8.05	37	-8.84	52
4	-8.20	43	-7.98	36	-8.60	43
5	-8.16	33	-8.30	37	-8.17	37
all	$\mathbf{-8.41} \pm 0.14$		$\mathbf{-8.35} \pm 0.15$		$\mathbf{-8.45} \pm 0.17$	

OAMe-G4 TIP4P

Supplementary Table 24: **OAMe-G4 with water model TIP4P binding free energy breakdown.** Binding free energy ΔG (kcal mol^{-1}) of ligand G1 and its corresponding statistical weight w (a.u.) in every simulation block of calculations using Deep-LDA CVs from three different training.

Block	CV s_w^a		CV s_w^b		CV s_w^c	
	ΔG	w	ΔG	w	ΔG	w
1	-3.92	70	-3.24	25	-4.02	44
2	-2.70	18	-4.89	60	-3.34	38
3	-3.83	57	-3.93	52	-4.46	36
4	-3.05	33	-3.59	39	-4.21	39
5	-3.26	42	-3.35	41	-3.82	38
all	$\mathbf{-3.54} \pm 0.23$		$\mathbf{-3.95} \pm 0.33$		$\mathbf{-3.97} \pm 0.19$	

OAMe-G5 TIP4P

Supplementary Table 25: **OAMe-G5 with water model TIP4P binding free energy breakdown.** Binding free energy ΔG (kcal mol^{-1}) of ligand G1 and its corresponding statistical weight w (a.u.) in every simulation block of calculations using Deep-LDA CVs from three different training.

Block	CV s_w^a		CV s_w^b		CV s_w^c	
	ΔG	w	ΔG	w	ΔG	w
1	-5.17	31	-5.40	45	-6.05	60
2	-5.73	52	-5.42	48	-5.81	31
3	-5.83	57	-6.02	49	-6.20	43
4	-5.14	35	-5.49	41	-5.42	41
5	-5.63	38	-5.93	40	-5.44	47
all	$\mathbf{-5.56} \pm 0.15$		$\mathbf{-5.65} \pm 0.14$		$\mathbf{-5.80} \pm 0.16$	

OAMe-G6 TIP4P

Supplementary Table 26: **OAMe-G6 with water model TIP4P binding free energy breakdown.** Binding free energy ΔG (kcal mol^{-1}) of ligand G1 and its corresponding statistical weight w (a.u.) in every simulation block of calculations using Deep-LDA CVs from three different training.

Block	CV s_w^a		CV s_w^b		CV s_w^c	
	ΔG	w	ΔG	w	ΔG	w
1	-6.05	45	-6.49	50	-5.70	49
2	-5.56	21	-5.78	48	-5.53	45
3	-6.03	43	-6.91	33	-5.21	21
4	-6.21	34	-5.51	29	-5.72	36
5	-6.00	54	-5.91	58	-6.26	31
all	$\mathbf{-6.01} \pm 0.09$		$\mathbf{-6.11} \pm 0.24$		$\mathbf{-5.70} \pm 0.16$	

Supplementary References

[1] Yin, J. *et al.* Overview of the SAMPL5 host–guest challenge: Are we doing better? *Journal of Computer-Aided Molecular Design* **31**, 1–19 (2017). URL http://link.springer.com/10.1007/s10822-016-9974-4.

[2] Yin, J., Henriksen, N. M., Slochower, D. R. & Gilson, M. K. The SAMPL5 host–guest challenge: computing binding free energies and enthalpies from explicit solvent simulations by the attach-pull-release (APR) method. *Journal of Computer-Aided Molecular Design* **31**, 133–145 (2017). URL http://link.springer.com/10.1007/s10822-016-9970-8.

[3] Bosisio, S., Mey, A. S. & Michel, J. Blinded predictions of host-guest standard free energies of binding in the SAMPL5 challenge. *Journal of Computer-Aided Molecular Design* **31**, 61–70 (2017).

[4] Bhakat, S. & Söderhjelm, P. Resolving the problem of trapped water in binding cavities: prediction of host–guest binding free energies in the SAMPL5 challenge by funnel metadynamics. *Journal of Computer-Aided Molecular Design* **31**, 119–132 (2017).

[5] Abraham, M. J. *et al.* GROMACS: High performance molecular simulations through multi-level parallelism from laptops to supercomputers. *SoftwareX* **1-2**, 19–25 (2015). URL https://linkinghub.elsevier.com/retrieve/pii/S2352711015000059.

[6] Tribello, G. A., Bonomi, M., Branduardi, D., Camilloni, C. & Bussi, G. PLUMED 2: New feathers for an old bird. *Computer Physics Communications* **185**, 604–613 (2014). URL http://linkinghub.elsevier.com/retrieve/pii/S0010465513003196.

[7] Paszke, A. *et al.* Automatic differentiation in PyTorch. *Adv. Neural Inf. Process. Syst.* **32**, 8024–8035 (2019).

[8] Wang, J., Wolf, R. M., Caldwell, J. W., Kollman, P. A. & Case, D. A. Development and testing of a general amber force field. *Journal of Computational Chemistry* **25**, 1157–1174 (2004). URL http://doi.wiley.com/10.1002/jcc.20035.

[9] Bayly, C. I., Cieplak, P., Cornell, W. & Kollman, P. A. A well-behaved electrostatic potential based method using charge restraints for deriving atomic charges: the RESP model. *The Journal of Physical Chemistry* **97**, 10269–10280 (1993). URL https://pubs.acs.org/doi/abs/10.1021/j100142a004.

[10] Jorgensen, W. L., Chandrasekhar, J., Madura, J. D., Impey, R. W. & Klein, M. L. Comparison of simple potential functions for simulating liquid water. *The Journal of Chemical Physics* **79**, 926–935 (1983). URL http://aip.scitation.org/doi/10.1063/1.445869.

[11] Bussi, G., Donadio, D. & Parrinello, M. Canonical sampling through velocity rescaling. *The Journal of Chemical Physics* **126**, 014101 (2007). URL http://aip.scitation.org/doi/10.1063/1.2408420.

[12] Bonati, L., Rizzi, V. & Parrinello, M. Data-Driven Collective Variables for Enhanced Sampling. *The Journal of Physical Chemistry Letters* 2998–3004 (2020). URL http://arxiv.org/abs/2002.06562https://pubs.acs.org/doi/10.1021/acs.jpclett.0c00535.

[13] Kingma, D. P. & Ba, J. Adam: A Method for Stochastic Optimization. In *ICLR*, 1–15 (2015). URL http://arxiv.org/abs/1412.6980.

[14] Limongelli, V., Bonomi, M. & Parrinello, M. Funnel metadynamics as accurate binding free-energy method. *Proceedings of the National Academy of Sciences* **110**, 6358–6363 (2013). URL http://www.pnas.org/cgi/doi/10.1073/pnas.1303186110.

[15] Pérez-Conesa, S., Piaggi, P. M. & Parrinello, M. A local fingerprint for hydrophobicity and hydrophilicity: From methane to peptides. *The Journal of Chemical Physics* **150**, 204103 (2019). URL http://dx.doi.org/10.1063/1.5088418http://aip.scitation.org/doi/10.1063/1.5088418.

[16] Invernizzi, M. & Parrinello, M. Rethinking Metadynamics: From Bias Potentials to Probability Distributions. *The Journal of Physical Chemistry Letters* **11**, 2731–2736 (2020). URL http://arxiv.org/abs/1909.07250https://pubs.acs.org/doi/10.1021/acs.jpclett.0c00497.

[17] Ewell, J., Gibb, B. C. & Rick, S. W. Water inside a hydrophobic cavitand molecule. *Journal of Physical Chemistry B* **112**, 10272–10279 (2008).

[18] Horn, H. W. *et al.* Development of an improved four-site water model for biomolecular simulations: TIP4P-Ew. *The Journal of Chemical Physics* **120**, 9665–9678 (2004). URL http://aip.scitation.org/doi/10.1063/1.1683075.
